# Lipid metabolism disorders and albuminuria risk: insights from National Health and Nutrition Examination Survey 2001–2018 and Mendelian randomization analyses

**DOI:** 10.1080/0886022X.2024.2420841

**Published:** 2024-11-03

**Authors:** Yangyang Wang, Sen Li

**Affiliations:** a Second Medical College of Wenzhou Medical University, Wenzhou, China; b School of Basic Medical Sciences, Wenzhou Medical University, Wenzhou, China

**Keywords:** Lipid metabolism disorder, Albuminuria, National Health and Nutrition Examination Survey, Mendelian randomization

## Abstract

**Background:**

Previous studies have revealed an underlying connection between abnormal lipid metabolism and albuminuria. We aim to investigate the causal relationship between lipid metabolism disorders and the risk of albuminuria from both a population and genetic perspective.

**Methods:**

A cross-sectional study was conducted by using data from the National Health and Nutrition Examination Survey (NHANES) 2001-2018. Multivariable-adjusted logistic regression, subgroup analysis, interaction tests and restricted cubic spline (RCS) were employed statistically. Mendelian randomization (MR) analysis was performed to validate the causal relationship between exposure and outcome to mitigate confounding factors and reverse causation interference.

**Results:**

After adjusting for confounders, HDL levels (1.03-2.07 nmol/L) were associated with a reduced risk of albuminuria. In contrast, elevated cholesterol levels (>6.2 nmol/L) and triglyceride levels (>2.3 nmol/L) were associated with an increased risk of albuminuria. Serum triglyceride concentration emerged as a potential risk factor for albuminuria. In MR analysis, a reduced risk of albuminuria was associated with serum total HDL level (IVW: OR = 0.91, 95% CI = 0.86-0.97, *p* = 0.002). In contrast, cholesterol esters in medium VLDL (IVW: OR = 1.05, 95% CI = 1.00-1.10, *p* = 0.032), chylomicrons and extremely large VLDL (IVW: OR = 1.08, 95% CI = 1.03-1.14, *p* = 0.003), and triglycerides (IVW: OR = 1.14, 95% CI = 1.09-1.19, *p* < 0.001) were associated with an increased risk of albuminuria.

**Conclusion:**

A causal relationship exists between serum lipid metabolism disorder and albuminuria risk. Further validation of additional blood lipid metabolism biomarkers is imperative for future studies.

## Introduction

Increasing or decreasing levels of serum lipids cause various health effects in the human body, which are called disorders. These types of disorders usually increase triglyceride, low-density lipoprotein cholesterol (LDL), or both lipid levels. The body requires the useful fatty acid high-density lipoprotein cholesterol (HDL), which helps to transport bad cholesterol out of the body. Similarly, the accumulation of bad and unwanted lipids, such as fatty LDLs and triglyceride, damage the arteries and the kidney, and has serious consequences for cardiovascular and renal health [1]. Recently, Xiao et al. [2] published an article on inherited complex lipid metabolism disorders, stating that over 80 diseases have been identified as complex lipid metabolism defects [2] with main consequences of chronic kidney disease (CKD) and a higher incidence of cardiovascular events [3,4]. For instance, fluctuations in levels of certain lipid metabolites, such as triglycerides and decreased HDL, may be associated with renal impairment [5,6]. Additionally, disrupted cholesterol balance in the body may induce the development of diabetes and exacerbate the risk of albuminuria [7,8]. Reduced HDL levels and elevated LDL and triglycerides are also significant risk factors for various cardiovascular diseases, such as coronary heart disease and apoplexy [9]. Moreover, dyslipidemia is a significant complication of CKD, and in severe renal failure patients, it may even lead to a further decreased levels of LDL and cholesterol [10].

The consequences of lipid metabolism disorders are complex. Identifying commonalities among them may be the key to preventing and solving these diseases. At this point, albuminuria appeared in the researchers’ field of view. Albuminuria, defined as a urinary albumin-to-creatinine ratio (UACR) greater than 30 mg/g, is often seen in patients with lipid metabolism disorders [11,12]. Within this range, a UACR of 30-300 is defined as microalbuminuria, while a UACR greater than 300 is defined as macroalbuminuria [13]. The researchers found that microalbuminuria can serve as an indicator of renal function impairment and a sensitive marker for the early progression of chronic kidney disease [14]. Further, the degree of albuminuria is closely related to the prognosis of renal conditions, elevated levels of albuminuria frequently indicate worsening of the disease and unfavorable results [15]. Meanwhile, albuminuria is also a potential biomarker for various cardiovascular diseases [16]. There is evidence indicating that albuminuria increases the risk of apoplexy and coronary heart disease [16]. It is worth noting that patients with lipid metabolism disorders may face an increased risk of albuminuria [17]. Obviously, albuminuria serves as a significant biomarker for cardiorenal diseases and exhibits intricate associations with lipid metabolism disorders. Additionally, the role of serum lipid metabolism levels in screening and treating albuminuria should also be taken into consideration. However, the causal relationship and specific mechanisms between lipid metabolism and albuminuria are not fully understood, and there is a lack of detailed research specifically linking the two fields.

In this study, the relationship between serum lipid metabolism and albuminuria was investigated by stratifying serum triglycerides, cholesterol, HDL, and LDL concentrations of participants in the National Health and Nutrition Examination Survey (NHANES). Two-sample Mendelian randomization (MR) was employed by using single-nucleotide polymorphisms (SNPs) from genome-wide association studies as instrumental variables (IVs) to simulate a randomized controlled trial (RCT) and investigate the unidirectional causal relationship between exposure and outcome [18]. In summary, we aim to investigate the causal relationship between lipid metabolism disorders and the risk of albuminuria from both a population and genetic perspective.

## Methods

### The study population in NHANES

NHANES are designed to assess the nutritional status, health conditions, and risk factors for diseases among the American population [19]. NHANES conducts face-to-face interviews, physical examinations, and laboratory tests on a nationally representative sample to gather various health and nutrition-related data, which are released biennially. These data are crucial for understanding dietary habits, disease prevalence, and epidemiological characteristics of chronic diseases among diverse racial, gender, and age groups [20]. Detailed information can be found on the website (https://www.cdc.gov/nchs/nhanes). All procedures are conducted following relevant guidelines and ethical regulations, with informed consent obtained from all participants.

**Figure 1.**
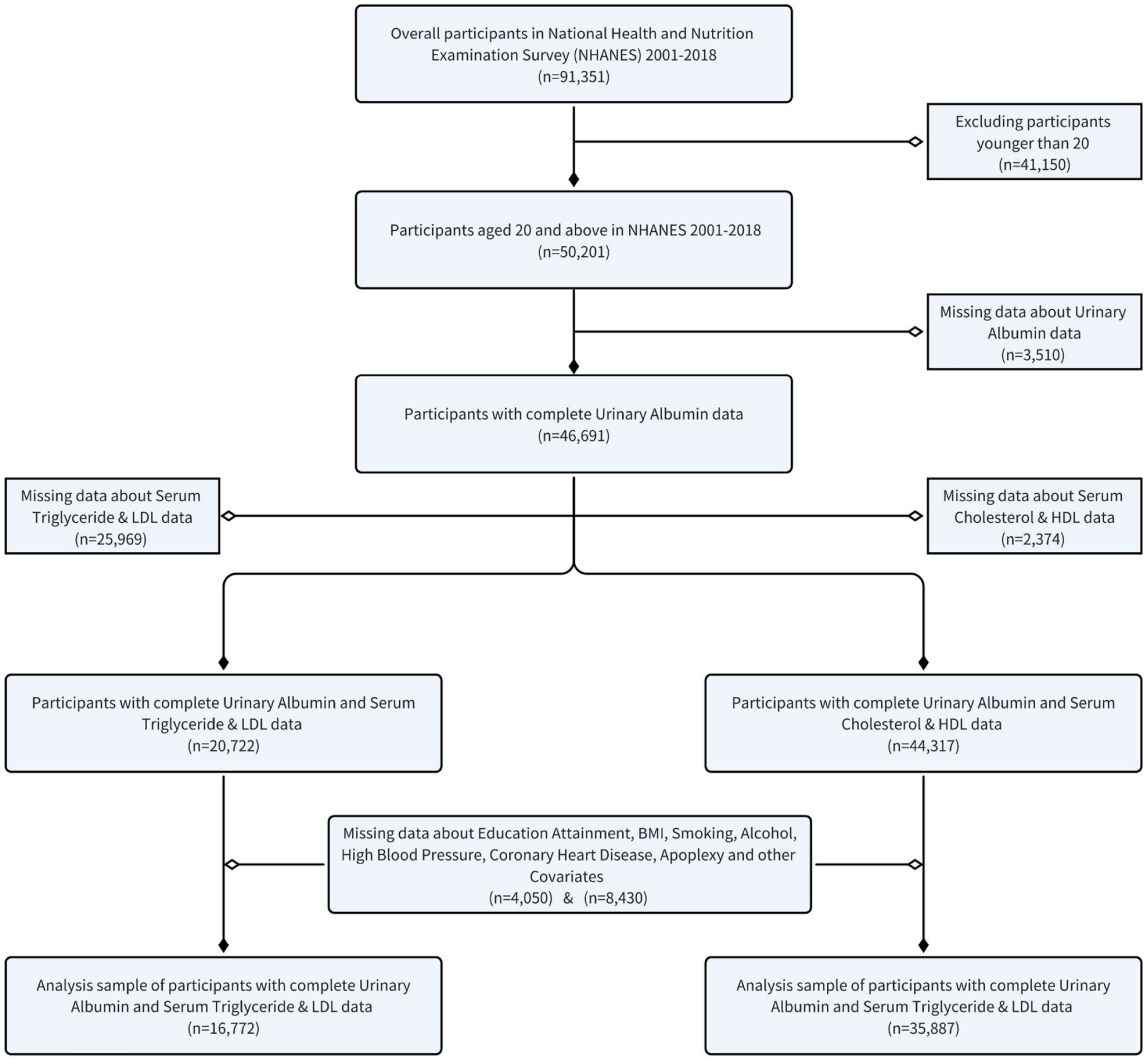
Study design in NHANES. Flowchart of sample selection from NHANES (2001–2018).

In this study, the population included statistical data from nine survey cycles spanning the years 2001 to 2018. Due to certain surveys or tests (such as alcohol consumption) being limited to individuals aged 20 and above, the analyzed sample was restricted to adults aged 20 years or older. Additionally, serum HDL and cholesterol concentrations, along with serum LDL and triglyceride concentrations, involved different questionnaires and populations requiring separate screenings. Figure 1 provides a detailed overview of the inclusion and exclusion processes of the study.

### Assessment of lipid metabolism levels and albuminuria in NHANES

NHANES quantitatively assesses serum triglycerides, cholesterol, HDL, LDL, urinary albumin concentration, and UACR using established laboratory methods. Serum triglycerides was measured by Roche Modular P chemistry analyzer with an enzymatic method (Glycerol phosphate oxidase). Serum total cholesterol is determined through a color reaction catalyzed by peroxidase, which generates H_2_O_2_ by the reactions of cholesterol esterase and cholesterol oxidase. Serum HDL concentration is directly measured using polyethylene glycol-coupled cholesterol esterase and cholesterol oxidase, alongside α-cyclodextrin sulfate to eliminate interference from Apolipoprotein B (APOB). Serum LDL concentration, calculated using the Friedewald formula after a minimum fast of 8.5 h, are based on the measured values of total cholesterol, HDL, and triglycerides ([LDL-cholesterol] = [total cholesterol] - [HDL-cholesterol] - [triglycerides/5]) [21]. Urinary albumin concentration is assessed through a noncompetitive sandwich fluorescence immunoassay method proposed by Chavers et al. [22]. Urinary creatinine measurements are performed using the Roche/Hitachi Modular P Chemistry Analyzer. The ‘NHANES Laboratory/Medical Technicians Procedures Manual’ offers detailed explanations of the biochemical analysis methods and laboratory quality assurance protocols.

We utilized 30 mg/g and 300 mg/g as the threshold values for grading urinary albumin concentration based on the definition of albuminuria. ‘<30 mg/g’ is defined as normal level, ‘30-300’ is classified as microalbuminuria, and ‘≥300’ is considered macroalbuminuria. In the regression model, albuminuria is included as a binary outcome variable. Further, we stratify serum triglycerides (‘<1.7′, ‘1.7-2.3′, ‘≥2.3’nmol/L), cholesterol (‘<5.2′, ‘5.2-6.2′, ‘≥6.2’nmol/L), HDL (‘<1.03′, ‘1.03-2.07′, ‘≥2.07’nmol/L), and LDL (‘<2.6′, ‘2.6-4.1′, ‘≥4.1’nmol/L) levels based on their corresponding thresholds to describe lipid metabolism disorders.

### Covariate definition in NHANES

On the basis of previous studies, a range of factors possibly associated with albuminuria, including age, age group (‘20-39′, ‘40-59′, ‘60-79′, ‘80+’), gender (male or female), race (‘Mexican American’, ‘Non-Hispanic Black’, ‘Non-Hispanic White’, ‘other Hispanic’, ‘other Race -Including Multi-Racial’), education level (‘Less Than 9th Grade’, ‘9-11th Grade (Includes 12th grade with no diploma)’, ‘High School Grad/GED or Equivalent’, ‘Some College or Associate of Arts degree’, ‘College Graduate or above’), body mass index (BMI) (‘Normal’, ‘Obese’, ‘Overweight’, ‘Underweight’), waist circumference, smoking (‘1-5 drinks/month’, ‘5-10 drinks/month’, ‘10+ drink/month’, ‘Non-drinker’), alcohol consumption (‘Current smoker’, ‘Former smoker’, ‘NO smoker’), hypertension, diabetes, coronary heart disease, and apoplexy were screened. Age group, gender, race, education attainment, BMI, smoking, alcohol consumption, hypertension, diabetes, coronary heart disease, and apoplexy were defined as categorical variables. Age, and waist circumference were defined as continuous variables. Physical examination data included BMI and waist circumference, while questionnaire data comprised hypertension, diabetes, coronary heart disease, and apoplexy [23–31].

### Genetically instrumental variables for serum lipid metabolism and albuminuria in MR

Genetic instrumental variables were selected from various genome-wide association studies (GWAS) to validate the causal relationship between exposure and outcome. In addition to serum levels of total triglycerides, cholesterol, HDL, and LDL, we also included more exposure indicators that can represent the level of serum lipid metabolism, such as cholesterol esters in large HDL, cholesterol esters in large VLDL, cholesterol esters in medium HDL, cholesterol esters in medium LDL, cholesterol esters in medium VLDL, chylomicrons, and extremely large VLDL particles [32–34]. The genetic variants related to serum lipid metabolism in these GWAS studies are derived from European populations. The GWAS summary data on albuminuria is based on a large meta-analysis (*n* = 348,954, including 51,861 cases and 297,093 ­controls), with detailed information available on CKDgen (https://ckdgen.imbi.uni-freiburg.de/datasets) [35]. Additionally, we utilized the GWAS data (*n* = 46,061) from the independent cohort for proteinuria conducted by Teumer et al. to validate the stability of the MR results [36].

**Figure 2.**
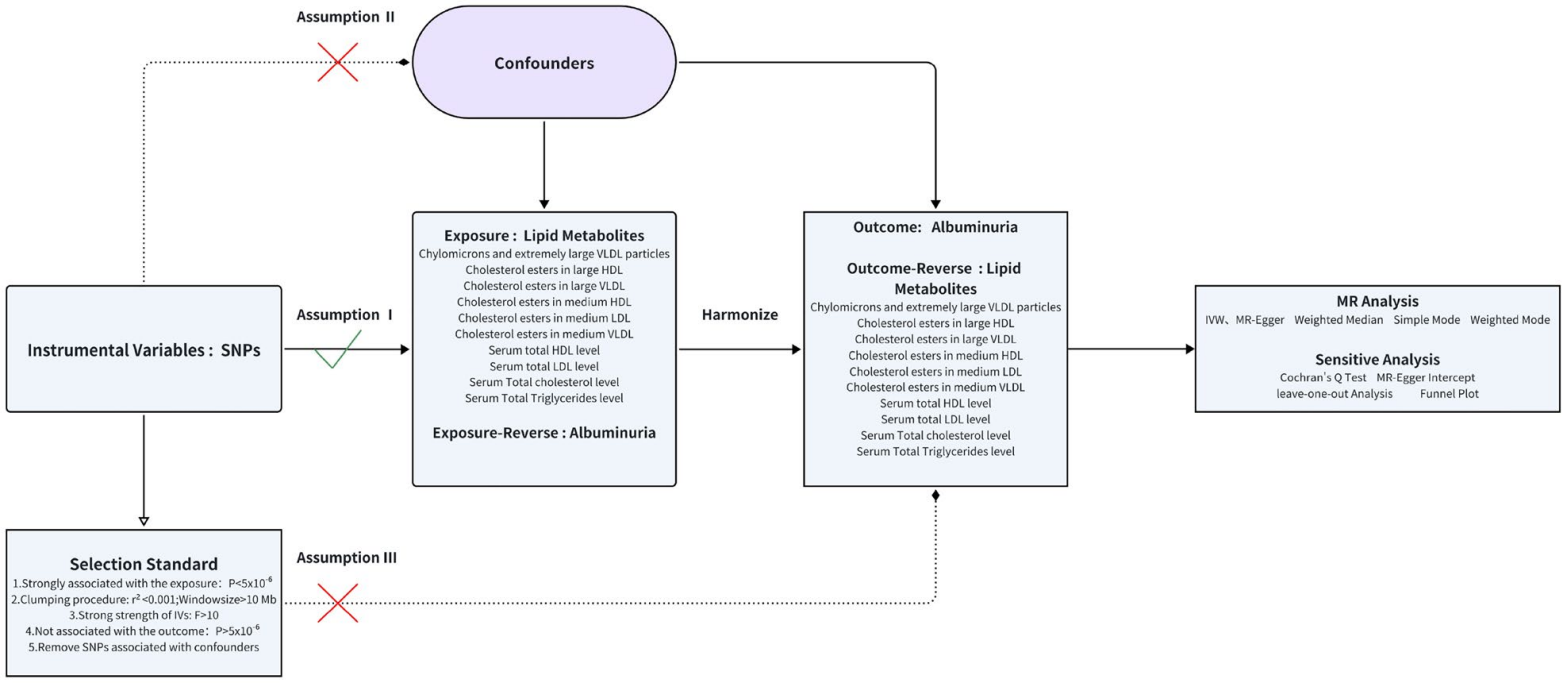
Study design in MR: Assumption I: IVs was strongly correlated with exposure. Assumption II: IVs was not associated with confounders. Assumption III: IVs was not associated with outcome.

Standardized quality assessment was employed to screen instrumental variables, ensuring the rigor and reliability of MR analysis. In order to improve the thoroughness of the results, a lenient genome-wide significance cutoff of *p* < 5 × 10^−6^ for the selection of SNPs linked to lipid metabolism exposure traits was utilized [37]. An evaluation of Linkage Disequilibrium (LD) to ensure the independence of the chosen instrumental variables (IVs), maintaining an LD r^2^ threshold of less than 0.001 within a 10 MB range, was carried out. The computation of the F-statistic was performed for each SNP, and those with F values below 10 were excluded to mitigate any potential bias arising from weak instruments. Throughout the standardization procedure, SNPs that were incompatible or displayed a palindromic structure with intermediate allele frequency were omitted [38–40]. In the Reverse-MR analysis, albuminuria was considered as the exposure factor and serum lipid metabolism indicators as the outcome, repeating the aforementioned process. Figure 2 illustrates the flowchart of MR and the three core assumptions of MR. We excluded IVs that had a causal relationship with the outcomes. Additionally, to mitigate potential confounding effects, we utilized PhenoScanner to examine the correlations among potential confounders related to the IVs.

### Statistical analyses

The concentrations of serum triglycerides, cholesterol, HDL, and LDL, and their corresponding grades were stratified to delineate the baseline characteristics of both the total population and participants in each stratum. Subsequently, three multi-factor weighted logistic regression models were developed for participants based on the aforementioned serum lipid concentrations and grades to explore the association between serum lipid metabolism and albuminuria. Continuous variables were described using the mean and standard error of the mean, while categorical variables were depicted using percentages. The normality of continuous variables was assessed through Kolmogorov-Smirnov testing and histograms. For normally distributed continuous variables, the Student’s t-test was employed; otherwise, the Wilcoxon rank-sum test was utilized. Between-group comparisons of categorical variables were conducted using the chi-square test and Fisher’s exact test. Given the utilization of NHANES data from nine survey cycles with intricate sampling designs, sample weights were recalibrated to bolster result reliability. Model 1 showcases an unadjusted multi-factor weighted logistic regression model. Model 2 expands on Model 1 by incorporating adjustments for age, gender, race, education attainment. Model 3 further enhances Model 2 by including additional adjustments for BMI, waist circumference, smoking, alcohol consumption, hypertension coronary, heart disease, apoplexy, and diabetes. Moreover, Subgroup analyses were conducted to examine the relationship between lipid metabolism and albuminuria. These analyses considered variables such as age group, gender, race, education level, BMI, diabetes, hypertension, apoplexy, and coronary heart disease. Additionally, interaction tests were employed to assess the consistency of this association across different subgroups. RCS plots were used to explore the potential nonlinear relationship between these factors.

In MR analysis, we consider the IVW method results as the primary analytical outcomes. Additionally, these results were validated by using MR-Egger, weighted median, simple mode, and weighted mode methods. In case of contradictory findings, the IVW results remain unaffected. Detailed descriptions of these methods have been provided in previous studies [41,42]. Cochran’s Q test was employed to assess the outcomes of IVW and MR-Egger analyses (*p* < 0.05 indicates heterogeneity). The MR-Egger intercept test was employed to examine the horizontal pleiotropy of SNPs, where the regression intercept assesses the magnitude of horizontal pleiotropy (*p* < 0.05 indicates potential pleiotropy). The IVW model, based on random effects, is utilized for the analysis, which effectively manages heterogeneity and ensures a more precise estimation of causal effects. Furthermore, we eliminated SNPs with an F-statistic below 10 to ensure the high quality and stability of the instrumental variables. Additionally, leave-one-out analysis and funnel plots were conducted for heterogeneity assessment [42]. The ‘TwoSampleMR’ package (version 0.5.7) in R (version 4.3.1) was utilized for MR analysis.

## Results

### Baseline characteristics of the study participants in NHANES

A total of 35,887 participants (including those with serum HDL and cholesterol) and 16,772 participants (including those with serum LDL and triglycerides) participated in the cross-sectional study. The baseline characteristics of the participants can be found in Table 1 and Table 2. Individuals with albuminuria often exhibited characteristics such as advanced age, male, lower educational attainment, obesity, increased waist circumference, smoking, diabetes, hypertension, and apoplexy (*p* < 0.001). This association intensifies with disease progression. No correlation was observed between serum cholesterol concentrations and albuminuria, leading to its exclusion from subsequent analyses.

**Table 1.** Baseline characteristics of the research population (cholesterol & HDL) with different types of albuminuria.

	Non-Albuminuria (31572)	Microalbuminuria (3580)	Macroalbuminuria (735)	Total (35887)	*P*-value
Age Group (%)					<0.001
20-39	11537(36.5%)	637(17.8%)	94(12.8%)	12268(34.2%)	
40-59	10473(33.2%)	933(26.1%)	208(28.3%)	11614(32.4%)	
60-79	7956(25.2%)	1425(39.8%)	317(43.1%)	9698(27.0%)	
80+	1606(5.1%)	585(16.3%)	116(15.8%)	2307(6.4%)	
Age					<0.001
Mean ± SD	48.11 ± 17.68	59.02 ± 17.95	60.58 ± 16.13	49.45 ± 18.04	
Sex (%)					<0.001
female	16101(51.0%)	1886(52.7%)	317(43.1%)	18304(51.0%)	
male	15471(49.0%)	1694(47.3%)	418(56.9%)	17583(49.0%)	
Race (%)					<0.001
Mexican American	5399(17.1%)	640(17.9%)	149(20.3%)	6188(17.2%)	
Other Hispanic	2618(8.3%)	285(8.0%)	66(9.0%)	2969(8.3%)	
Non-Hispanic White	14909(47.2%)	1603(44.8%)	246(33.5%)	16758(46.7%)	
Non-Hispanic Black	6096(19.3%)	807(22.5%)	212(28.8%)	7115(19.8%)	
Other Race -Including Multi-Racial	2550(8.1%)	245(6.8%)	62(8.4%)	2857(8.0%)	
Education Attainment (%)					<0.001
Less Than 9^th^ Grade	3305(10.5%)	621(17.3%)	146(19.9%)	4072(11.3%)	
9-11^th^ Grade (Includes 12^th^ grade with no diploma)	4464(14.1%)	639(17.8%)	148(20.1%)	5251(14.6%)	
High School Grad/GED or Equivalent	7288(23.1%)	823(23.0%)	168(22.9%)	8279(23.1%)	
Some College or AA degree	9201(29.1%)	947(26.5%)	176(23.9%)	10324(28.8%)	
College Graduate or above	7314(23.2%)	550(15.4%)	97(13.2%)	7961(22.2%)	
BMI Group (%)					<0.001
Normal	8898(28.4%)	841(23.5%)	172(23.4%)	9982(27.8%)	
Obese	11185(35.4%)	1551(43.3%)	346(47.1%)	13082(36.5%)	
Overweight	10976(34.8%)	1093(30.5%)	207(28.2%)	12276(34.2%)	
Underweight	442(1.4%)	95(2.7%)	10(1.4%)	547(1.5%)	
Waistline (cm)					<0.001
Mean ± SD	98.39 ± 15.69	102.69 ± 17.13	105.18 ± 17.53	98.95 ± 15.95	
Smoking Status (%)					<0.001
Current smoker	6692(21.2%)	747(20 9%)	153(20.8%)	7592(21.2%)	
Former smoker	7702(24.4%)	1082(30.2%)	237(32.2%)	9021(25.1%)	
NO smoker	17178(54.4%)	1751(48.9%)	345(46.9%)	19274(53.7%)	
Alcohol Group (%)					<0.001
1-5 drinks/month	15623(49.5%)	1685(47.1%)	357(48.6%)	17665(492%)	
5-10 drinks/month	2481(7.9%)	214(6.0%)	41(5.6%)	2736(7.6%)	
10+ drink/month	4475(14.2%)	461(12.9%)	68(9.3%)	5004(13.9%)	
Non-drinker	8993(28.5%)	1220(34.1%)	269(36.6%)	10482(29.2%)	
Hypertension (%)					<0.001
FALSE	21606(68.4%)	1595(44.6%)	198(26.9%)	23399(65.2%)	
TRUE	9966(31.6%)	1985(55.4%)	537(73.1%)	12488(34.8%)	
Coronary Heart Disease (%)					<0.001
FALSE	30502(96.6%)	3305(92.3%)	649(88.3%)	34658(96.6%)	
TRUE	1070(3.4%)	275(7.7%)	86(11.7%)	1229(3.4%)	
Apoplexy (%)					<0.001
FALSE	30704(97.3%)	3935(93.0%)	675(89.4%)	34659(96.6%)	
TRUE	868(2.7%)	298(7.0%)	80(10.6%)	1229(3.4%)	
Diabetes (%)					<0.001
FALSE	28734(91.0%)	2567(71.7%)	353(48.0%)	31654(88.2%)	
TRUE	2838(9.0%)	1013(28.3%)	382(52.0%)	4233(11.8%)	
Serum HDL Concentration (nmol/L)					<0.001
Mean ± SD	1.38 ± 0.42	1.36 ± 0.46	1.30 ± 0.42	1.38 ± 0.42	
Serum HDL Classification (%)					<0.001
<1.03 (nmol/L)	5839(18.5%)	803(22.4%)	190(25.9%)	6832(19.0%)	
1.03-2.07 (nmol/L)	23563(74.6%)	2527(70.6%)	509(69.3%)	26599(74.1%)	
≥2.07 (nmol/L)	2170(6.9%)	250(7.0%)	36(4.9%)	2456(6.8%)	
Serum Cholesterol Concentration (nmol/L)					0.428
Mean ± SD	5.08 ± 1.08	5.07 ± 1.21	5.12 ± 1.40	5.08 ± 1.10	
Serum Cholesterol Classification (%)					0.007
<5.2 (nmol/L)	18059(57.2%)	2080(58.1%)	412(56.1%)	20551(57.3%)	
5.2-6.2 (nmol/L)	9050(28.7%)	948(26.5%)	198(26.9%)	10196(28.4%)	
≥6.2 (nmol/L)	4463(14.1%)	552(15.4%)	125(17.0%)	5140(14.3%)	

**Table 2.** Baseline characteristics of the research population (triglycerides & LDL) with different types of albuminuria.

	Non-Albuminuria (14700)	Microalbuminuria (1718)	Macroalbuminuria (354)	Total (16772)	*P*-value
Age Group (%)					<0.001
20-39	5304(36.1%)	293(17.1%)	47(13.3%)	5644(33.7%)	
40-59	4866(33.1%)	425(24.7%)	97(27.4%)	5388(32.1%)	
60-79	3784(25.7%)	703(40.9%)	147(41.5%)	4634(27.6%)	
80+	746(5.1%)	297(17.3%)	63(17.8%)	1106(6.6%)	
Age					<0.001
Mean ± SD	48.32 ± 17.67	59.69 ± 18.04	60.87 ± 16.25	49.75 ± 18.09	
Sex (%)					0.011
female	7540(51.3%)	919(53.5%)	159(44.9%)	8618(51.4%)	
male	7160(48.7%)	799(46.5%)	195(55.1%)	8154(48.6%)	
Race (%)					<0.001
Mexican American	2473(16.8%)	313(18.2%)	73(20.6%)	2859(17.0%)	
Other Hispanic	1262(8.6%)	130(7.6%)	38(10.7%)	1430(8.5%)	
Non-Hispanic White	6991 (47.6%)	791(46.0%)	111(31.4%)	7893(47.1%)	
Non-Hispanic Black	2797(19.0%)	369(21.5%)	104(29.4%)	3270(19.5%)	
Other Race -Including Multi-Racial	1177(8.0%)	115(6.7%)	28(7.9%)	1320(7.9%)	
Education Attainment (%)					<0.001
Less Than 9^th^ Grade	1532(10.4%)	308(17.9%)	66(18.6%)	1906(11.4%)	
9-11^th^ Grade (Includes 12^th^ grade with no diploma)	2105(14.3%)	309(18.0%)	70(19.8%)	2484(14.8%)	
High School Grad/GED or Equivalent	3354(22.8%)	403(23.5%)	91(25.7%)	3848(22.9%)	
Some College or AA degree	4241(28.9%)	430(25.0%)	83(23.4%)	4754(28.3%)	
College Graduate or above	3468(23.6%)	268(15.6%)	44(12.4%)	3780(22.5%)	
BMI Group (%)					<0.001
Normal	4269(29.0%)	418(24.3%)	90(25.4%)	4777(28.5%)	
Obese	5109(34.8%)	734(42.7%)	163(46.0%)	6006(35.8%)	
Overweight	5099(34.7%)	528(30.7%)	95(26.8%)	5722(34.1%)	
Underweight	223(1.5%)	38(2.2%)	6(1.7%)	267(1.6%)	
Waistline(cm)					<0.001
Mean ± SD	98.22 ± 15.68	102.62 ± 16.99	105.23 ± 17.80	98.81 ± 15.94	
Smoking Status (%)					<0.001
Current smoker	3053(20.8%)	338(19.7%)	76 (21.5%)	3467(20.7%)	
Former smoker	3664(24.9%)	528(30.7%)	76 113(31.9%	4305(25.7%)	
NO smoker	7983(54.3%)	852(49.6%)	76 165(46.6%)	9000(53.7%)	
Alcohol Group (%)					<0.001
1-5 drinks/month	7285 (49.6%)	822(47.8%)	172(48.6%)	8279(49 4%)	
5-10 drinks/month	1132(7.7%)	90(5.2%)	22(6.2%)	1244(7.4%)	
10+ drink/month	2099(14.3%)	218(12.7%)	28(7.9%)	2345(14.0%)	
Non-drinker	4184(28.5%)	588(34.2%)	132(37.3%)	4904(29.2%)	
Hypertension (%)					<0.001
FALSE	9981 (67.9%)	748(43.5%)	91(25.7%)	10820(64.5%)	
TRUE	4719(32.1%)	970(56.5%)	263(74.3%)	5952(35.5%)	
Coronary Heart Disease (%)					<0.001
FALSE	14204(96.6%)	1564(91.0%)	307(86.7%)	16075(95.8%)	
TRUE	496(3.4%)	154(9.0%)	47(13.3%)	697(4.2%)	
Apoplexy (%)					<0.001
FALSE	14275(97.1%)	1583(92.1%)	318(89.8%)	16176(96.4%)	
TRUE	425(2.9%)	135(7.9%)	36(10.2%)	596(3.6%)	
Diabetes (%)					<0.001
FALSE	13418(91.3%)	1262(73.5%)	168(47.5%)	14848(88.5%)	
TRUE	1282(8.7%)	456(26.5%)	186(52.5%	1924(11.5%)	
Serum LDL Concentration (nmol/L)					<0.001
Mean ± SD	2.99 ± 0.91	2.92 ± 0.98	2.87 ± 1.08	2.98 ± 0.92	
Serum LDL Classification (%)					<0.001
<2.60 (nmol/L)	5214(35.5%)	692(40.3%)	163(46.0%)	6069(36.2%)	
2.60-4.10 (nmol/L)	7839(53.3%)	818(47.6%)	150(42.4%)	1896(11.3%)	
≥4.10 (nmol/L)	1647(11.2%)	208(12.1%)	41 (11.6%)	8807(52.5%)	
Serum Triglycerides Concentration (nmol/L)					<0.001
Mean ± SD	1.38 ± 0.76	1.52 ± 0.81	1.65 ± 0.87	1.40 ± 0.77	
Serum Triglycerides Classification (%)					<0.001
<1.7 (nmol/L)	10838(73.7%)	1156(67.3%)	220(62.1%)	12214(72.8%)	
1.7-2.3 (nmol/L)	2106(14.3%)	262(15.3%)	58(16.4%)	2426(14.5%)	
≥2.3 (nmol/L)	1756(11.9%)	300(17.5%)	76(21.5%)	2132(12.7%)	

### Lipid metabolism and albuminuria risk in NHANES

Serum lipid and apolipoprotein concentrations were explored as continuous or categorical variables to investigate their relationship with albuminuria. Regardless of model adjustments, higher levels of HDL (1.03-2.07 nmol/L) were associated with a decreased risk of albuminuria (OR = 0.85, 95% CI = 0.76-0.96, *p* = 0.014). Conversely, elevated cholesterol levels (>6.2 nmol/L) were associated with an increased risk of albuminuria (OR = 1.29, 95% CI = 1.13-1.47, *p* < 0.001). A higher risk of albuminuria was observed in those with high triglyceride levels (>2.3 nmol/L) (OR = 1.45, 95% CI = 1.22-1.72, *p* < 0.001). Additionally, serum triglyceride concentration (nmol/L) emerged as a potential risk factor for albuminuria (OR = 1.14, 95% CI = 1.04-1.25, *p* = 0.004). Based on Model 2, we found a negative correlation between higher LDL levels (2.6-4.1 nmol/L) (OR = 0.79, 95% CI = 0.68-0.92, *p* = 0.003) and LDL concentration (nmol/L) (OR = 0.92, 95% CI = 0.86-0.99, *p* = 0.034) with the risk of albuminuria. However, after fully adjusting for covariates, this relationship became non-significant (*p* > 0.05). The association between blood lipid metabolism and albuminuria is detailed in Table 3.

**Table 3.** Associations between lipid metabolism and albuminuria risk.

	Model 1, OR (95% CI, P)	Model 2, OR (95% CI, P)	Model 3, OR (95% CI, P)
Serum HDL Concentration (nmol/L)	0.87 (0.76-1.01) *p* = 0.07	0.90 (0.76-1.05) *p* = 0.199	0.99 (0.84-1.16) *p* = 0.887
Serum HDL Classification (nmol/L)			
<1.03	Reference	Reference	Reference
1.03-2.07	0.78 (0.70-0.88) *p* < 0.001	0.81 (0.72-0.91) *p* < 0.001	0.85 (0.76-0.96) *p* = 0.014
≥2.07	0.86 (0.70-1.06) *p* = 0.173	0.87 (0.69-1.08) *p* = 0.223	0.96 (0.76-1.20) *p* = 0.728
Serum Cholesterol Classification (nmol/L)			
<5.2	Reference	Reference	Reference
5.2-6.2	0.91 (0.83-1.01) *p* = 0.081	0.93 (0.84-1.03) *p* = 0.197	1.03 (0.93-1.14) *p* = 0.531
≥6.2	1.18 (1.05-1.32) *p* = 0.005	1.16 (1.01-1.32) *p* = 0.024	1.29 (1.13-1.47) *p* < 0.001
Serum LDL Concentration (nmol/L)	0.89 (0.83-0.96) *p* = 0.002	0.92 (0.86-0.99) *p* = 0.034	1.01 (0.93-1.08) *p* = 0.786
Serum LDL Classification (nmol/L)			
<2.6	Reference	Reference	Reference
2.6-4.1	0.76 (0.66-0.87) *p* < 0.001	0.79 (0.68-0.92) *p* = 0.003	0.92 (0.79-1.08) *p* = 0.363
≥4.1	0.87 (0.72-1.06) *p* = 0.183	0.90 (0.74-1.10) *p* = 0.323	1.06 (0.86-1.30) *p* = 0.559
Serum Triglyceride Concentration (nmol/L)	1.29 (1.19-1.40) *p* < 0.001	1.17 (1.07-1.29) *p* < 0.001	1.14 (1.04-1.25) *p* = 0.004
Serum Triglyceride Classification (nmol/L)			
<1.7	Reference	Reference	Reference
1.7-2.3	1.20 (0.98- 1.47) *p* = 0.066	1.02 (0.82-1.27) *p* = 0.804	0.99 (0.80- 1.22) *p* = 0.934
≥2.3	1.76 (1.51-2.05) *p* < 0.001	1.52 (1.27-1.82) *p* < 0.001	1.45 (1.22-1.72) *p* < 0.001

Logistic regression models.

Model 1: No covariates were adjusted.

Model 2: Adjusted for age, gender, race, education attainment.

Model 3: Adjusted for age, gender, race, education attainment, BMI, waist circumference, smoking, alcohol consumption, hypertension, coronary heart disease, apoplexy, and diabetes.

The results of the subgroup analysis showed significant associations between higher HDL levels (1.03-2.07 nmol/L) and albuminuria in certain subgroups, including females, age groups, education attainment, obese, hypertension, coronary heart disease, apoplexy, and diabetes. Significant associations were found between elevated triglyceride levels (≥ 2.3 nmol/L) and triglyceride concentration (nmol/L) across all subgroups. Interaction tests also proved the stability of the above associations (*p* > 0.05). However, the association of high cholesterol level (≥ 6.2 nmol/L) is influenced by age, race, and diabetes (*p* < 0.05). Figures S1–S4 illustrate more detailed sub-group analysis results.

**Figure 3.**
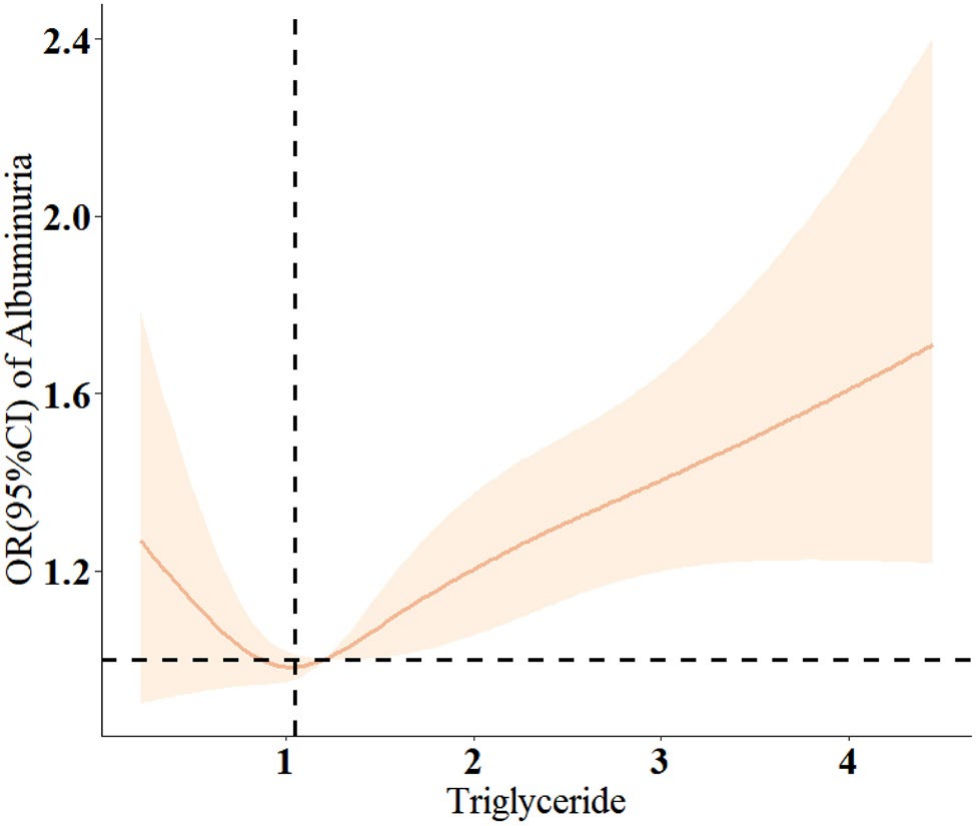
Restricted cubic spline (RCS) analysis of triglyceride and odds ratio of albuminuria based on Model 3.

**Figure 4.**
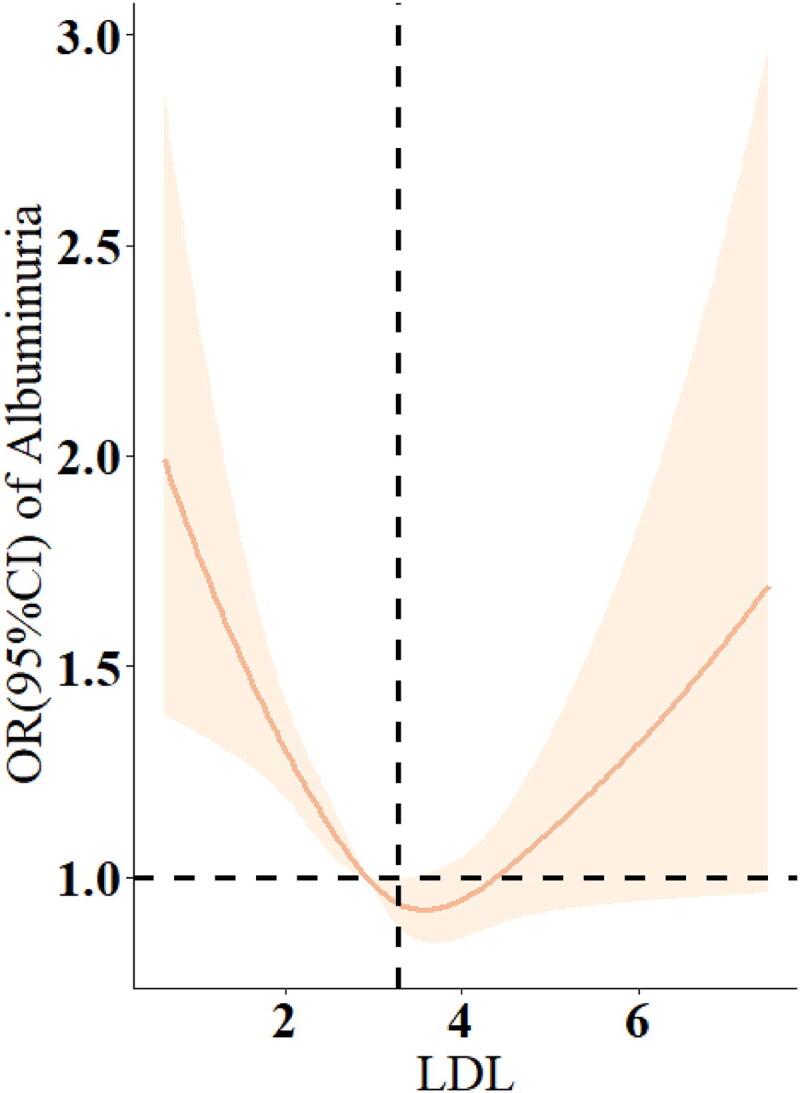
Restricted cubic spline (RCS) analysis of LDL and odds ratio of albuminuria based on Model 2.

The RCS plots revealed that there is no significant nonlinear relationship between triglyceride concentration and the risk of albuminuria (based on Model 3, *p* > 0.05) (Figure 3). However, a U-shaped relationship exists between LDL concentration and the risk of albuminuria (based on Model 2, *p* < 0.001), with a threshold point identified at 3.27 nmol/L (Figure 4).

### The causal association between lipid metabolism and albuminuria risk in MR

We identified a series of SNPs related to serum lipid metabolism (Cholesterol esters in large HDL, Cholesterol esters in large VLDL, Cholesterol esters in medium HDL, Cholesterol esters in medium LDL, Cholesterol esters in medium VLDL, chylomicrons, and extremely large VLDL particles). Detailed information provided in Table S1.

**Figure 5.**
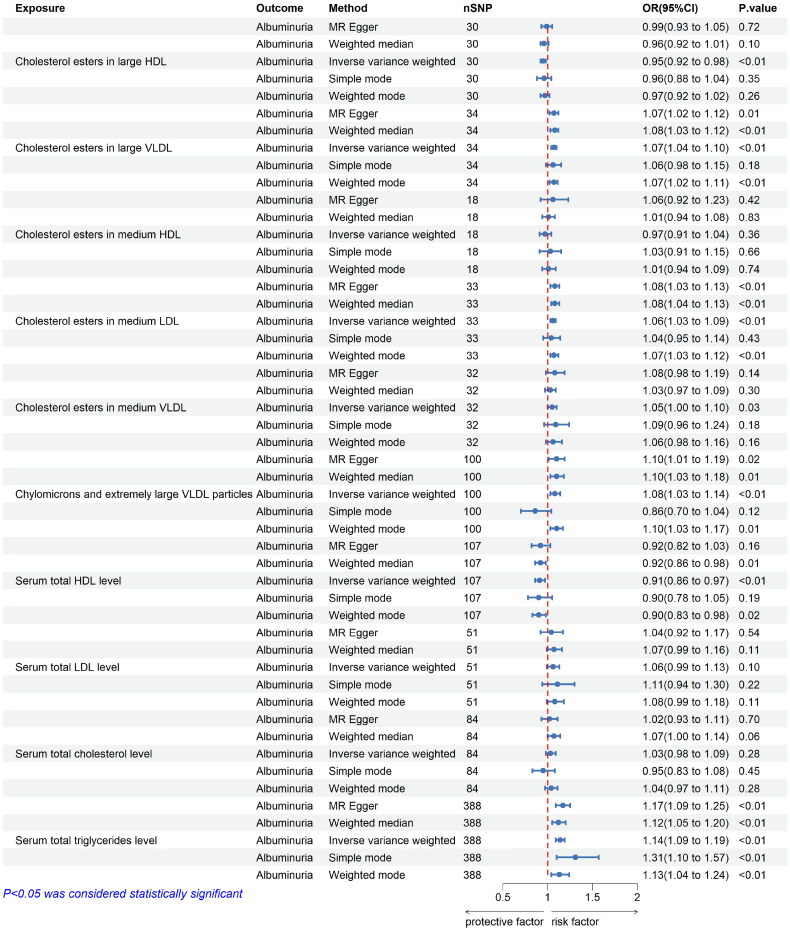
Causal estimates of lipid metabolism on albuminuria by MR analysis. Causal estimates of Lipid metabolism on Albuminuria by MR analysis (IVW, MR-Egger, Simple mode, Weight mode, Weight median). (A) Forest plots showing causal estimates of Lipid metabolism on Albuminuria. (B) Forest plots showing causal effects of Lipid metabolism on Albuminuria. The odds ratio (OR) was estimated using the fixed effect IVW method. The horizontal bars represent 95% confidence intervals (CI).

The MR analysis results indicate that Cholesterol esters in large HDL (IVW: OR = 0.95, 95% CI = 0.92-0.98, *p* = 0.003) and Serum total HDL level (IVW: OR = 0.91, 95% CI = 0.86-0.97, *p* = 0.002) are potential protective factors for MA. Conversely, Cholesterol esters in large VLDL (IVW: OR = 1.07, 95% CI = 1.04-1.10, *p* < 0.001), Cholesterol esters in medium LDL (IVW: OR = 1.06, 95% CI = 1.03-1.09, *p* < 0.001), Cholesterol esters in medium VLDL (IVW: OR = 1.05, 95% CI = 1.00-1.10, *p* = 0.032), Chylomicrons and extremely large VLDL particles (IVW: OR = 1.08, 95% CI = 1.03-1.14, *p* = 0.003), and Triglycerides (IVW: OR = 1.14, 95% CI = 1.09-1.19, *p* < 0.001) pose risks for albuminuria. No causal relationship was found between Serum total LDL level, Serum Total cholesterol level, and albuminuria (Figure 5). The findings are further supported by scatter plots, providing additional evidence. Detailed information provided in Figures S5, S6 and Table S2.

**Figure 6.**
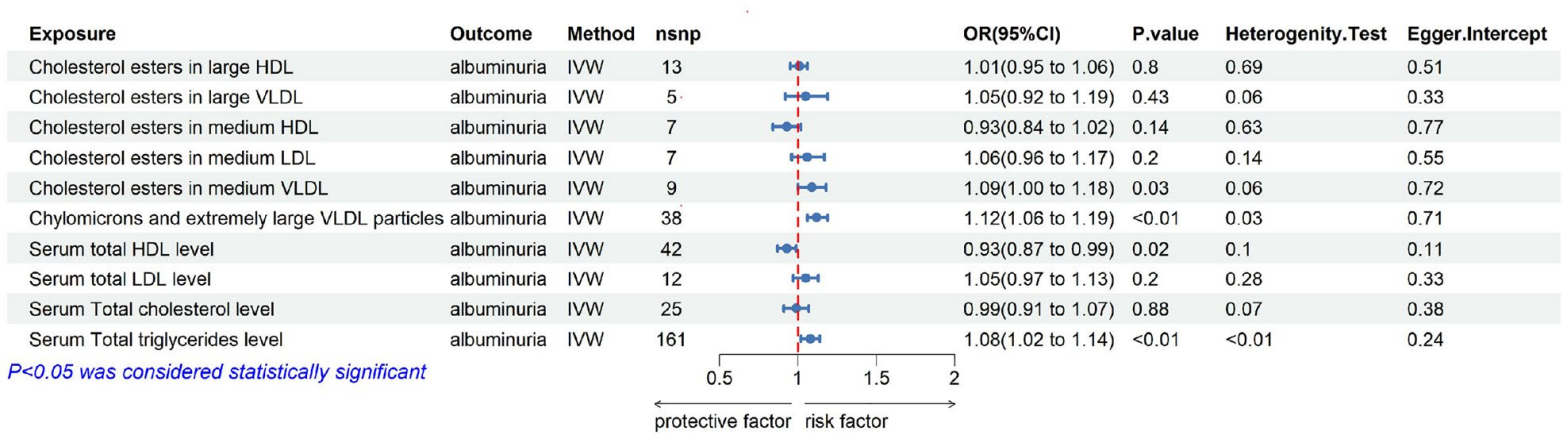
Causal estimates of lipid metabolism on albuminuria by MR analysis in independent cohort. Causal estimates of Lipid metabolism on Albuminuria by MR analysis (IVW, MR-Egger, Simple mode, Weight mode, Weight median). (A) Forest plots showing causal estimates of Lipid metabolism on Albuminuria. (B) Forest plots showing causal effects of Lipid metabolism on Albuminuria. The odds ratio (OR) was estimated using the fixed effect IVW method. The horizontal bars represent 95% confidence intervals (CI). (C) The Heterogeneity Tests represent the results of the IVW and MR-Egger analyses by Cochran’s Q test. The MR-Egger Intercept tests represent Horizontal pleiotropy.

Further, some exposure indicators analyzed in this study exhibited heterogeneity, yet directional pleiotropy was not present, thus not affecting the stability of the IVW results (Table S3). Funnel plots and leave-one-out plots further validate the above findings. Detailed information provided in Figures S7–S10.

In the reverse Mendelian randomization analysis, we did not identify an association between MA and the included serum metabolic markers, demonstrating a unidirectional causal relationship between exposure and outcome. Detailed information provided in Table S4.

Following validation with the independent cohort, we found that cholesterol esters in medium VLDL (IVW: OR = 1.09, 95% CI = 1.00-1.18, *p* = 0.03), chylomicrons and extremely large VLDL particles (IVW: OR = 1.12, 95% CI = 1.06-1.19, *p* < 0.01), triglycerides (IVW: OR = 1.08, 95% CI = 1.02-1.14, *p* < 0.01), and serum total HDL levels (IVW: OR = 0.93, 95% CI = 0.87-0.99, *p* = 0.02) are causally associated with the risk of albuminuria (Figure 6).

## Discussion

In this study, a cross-sectional analysis was conducted using data from the nationally representative NHANES 2001-2018 in the United States. Two-sample MR was employed to examine the potential causal relationship between lipid metabolism and the risk of albuminuria. The findings revealed that several lipid markers are positively associated with albuminuria risk. Specifically, elevated serum triglyceride concentration, extremely large VLDL particles, and cholesterol esters in medium VLDL particles showed a positive correlation with albuminuria risk. On the other hand, it was observed that serum HDL concentration displayed a potential protective effect against albuminuria. However, no significant correlation was found between serum total cholesterol or LDL concentrations and albuminuria.

The relationship between lipid metabolism and albuminuria has long been a subject of considerable interest. Some researchers suggest a close association between the triglyceride-glucose (Ty-G) index and urinary albumin concentration [43]. Evidence suggests a potential association between the urinary albumin-creatinine ratio and visceral obesity index [44]. A cohort study revealed that higher serum triglyceride levels are associated with a decline in renal glomerular filtration rate (GFR) and an increased incidence of cardiovascular diseases, accompanied by albuminuria [45]. Lipid metabolites play a crucial role as important cellular signaling molecules in the pathogenesis and progression of cardiorenal diseases. Lipid metabolism abnormalities commonly lead to lipid accumulation and metabolic dysfunction in renal diseases, subsequently triggering pathological processes such as oxidative stress, inflammation, and tissue damage [46–48]. For instance, elevated levels of triglycerides, an important lipid metabolite, are closely associated with renal dysfunction, glomerular injury, and increased albuminuria in renal diseases [49]. Further, abnormal triglyceride accumulation can contribute to oxidative stress and inflammatory responses within the kidneys [50]. Inflammatory factors play a critical role in the pathogenesis of renal injury and albuminuria [51]. Research indicates that a chronic inflammatory state may promote damage to renal tubules and glomeruli through various pathways [52]. For example, elevated levels of tumor necrosis factor-alpha (TNF-α) and interleukin-6 (IL-6) are closely associated with renal fibrosis, renal function decline, and the occurrence of albuminuria [53–55]. The presence of these inflammatory factors can not only directly damage renal cells but may also induce apoptosis and increase oxidative stress, leading to enhanced permeability of renal tubules and subsequent albumin leakage [56]. Elevated levels of triglycerides are also associated with an increased risk of cardiovascular diseases, such as atherosclerosis and coronary heart disease [57]. In this study, we found a positive correlation between triglyceride levels and albuminuria, regardless of whether triglycerides were examined as continuous or outcome variables. Moreover, MR analysis provided robust evidence at the genetic level, confirming and strengthening the causal relationship between triglycerides and albuminuria.

In addition, VLDL and LDL may have adverse effects in kidney diseases. Elevated levels of VLDL and LDL are associated with glomerular lesions, interstitial fibrosis, and tubular cell damage, which can intensify the progression of kidney diseases and lead to albumin leakage into the urine [58,59]. Moreover, increased levels of VLDL and LDL may induce and aggravate renal inflammation, activating inflammatory cells and promoting immune cell migration [58]. Different types of immune cells exhibit specific functions during the inflammatory response [60]. For instance, infiltration of monocytes and macrophages in the kidneys is significantly associated with an increase in albuminuria [61,62]. Activated macrophages release various cytokines and chemokines, further promoting inflammation and cellular damage [62]. Additionally, aberrant activation of T cells can intensify the inflammatory response, accelerating damage to renal tubules and glomeruli, ultimately resulting in albuminuria [63]. These lipoproteins are also associated with an increased risk of atherosclerosis and coronary heart disease [64]. In this study, we did not find a stable genetic association between serum LDL levels and the risk of albuminuria. Additionally, we found that certain metabolites associated with a series of VLDL (extremely large VLDL particles and cholesterol esters in medium VLDL particles) were related to the risk of albuminuria. The findings provide detailed insights into the complex correlations between specific lipid metabolites and albuminuria.

HDL, with its reverse cholesterol transport function, facilitates the clearance of cholesterol from arterial walls and its transport to the liver for metabolism, thus lowering the risk of atherosclerosis and coronary heart disease [65]. However, the role of HDL in kidney diseases is more complex. While HDL is generally considered to have a protective effect on the glomerular filtration barrier, in specific kidney diseases, elevated HDL levels may be associated with increased oxidative stress and inflammation in the glomerular basement membrane, leading to albumin leakage [66]. Several studies have suggested that higher HDL levels may be correlated with the activation of inflammatory cells and increased inflammation in kidney diseases [67]. In contrast, MR analysis has provided evidence of a negative correlation between HDL and the risk of albuminuria, further highlighting the causal relationship between HDL and albuminuria.

Some researchers argue that serum total cholesterol is a significant risk factor for albuminuria, however, at the genetic level, our view is different, as we found no significant association between serum total cholesterol and the risk of albuminuria [68]. We presume this may be attributed to the fact that they restricted their study population to individuals in the prediabetic stage.

It is noteworthy that there is an interplay between genetic and environmental factors in the relationship between lipid metabolism and albuminuria [69]. In this study, we found differences in the risk of albuminuria associated with lipid metabolism disorders among different racial subgroups, particularly in Non-Hispanic Black individuals who exhibited an increased risk of developing the condition. Further, factors such as gender, age, education level, BMI, and the presence of comorbidities (e.g. hypertension, coronary heart disease, stroke, diabetes) also influence the individual’s susceptibility to albuminuria risk in the context of lipid metabolism disorders. In clinical management, maintaining a healthy lipid metabolism may help prevent or alleviate the occurrence of microalbuminuria and related kidney diseases [70]. Based on our study, regular assessment of lipid levels, including triglycerides, HDL, and VLDL, is vital for the early identification of lipid metabolism disorders that may contribute to the risk of albuminuria and related kidney diseases. Early detection and monitoring of potential renal impairment through urine tests for urinary albumin levels can facilitate timely intervention to prevent or mitigate the progression of cardiorenal diseases. Given the differences in lipid metabolism among individual patients, personalized therapy plays a crucial role in clinical management. Based on our study, we recommend the implementation of routine screening and monitoring in clinical practice to ensure the identification of lipid metabolism disorders and early-stage albuminuria risk. Regular assessment and treatment of patients’ lipid levels is vital, particularly for individuals with high-risk factors such as obesity, diabetes, and hypertension [71]. Simultaneously, clinicians should consider individualized treatment strategies to address specific lipid abnormalities and mitigate the risk of albuminuria and renal function deterioration. For individuals with existing lipid abnormalities, timely pharmacological treatment, such as statins or fibrates, should be considered to effectively control lipid levels and reduce the risks of albuminuria and related cardiorenal diseases. Additionally, urine tests for monitoring urinary albumin levels can facilitate the early detection of potential renal impairment. Given the relative inconvenience of clinical urinary albumin testing, utilizing lipid levels as a significant biomarker for early kidney damage offers a more convenient method to evaluate patients’ health status [72]. Furthermore, comprehensive intervention measures are essential for improving patients’ overall health. Effective strategies can improve lipid metabolism levels. For patients with existing lipid abnormalities, timely pharmacological treatment, such as statins or fibrates, should be considered to effectively control lipid levels [73]. These measures can reduce the risks of albuminuria and renal function deterioration.

This study has several strengths: a cross-sectional was conducted study using NHANES 2001-2018 data, leveraging the generalizability and large sample size of NHANES to derive more stable and reliable results. NHANES studies alone cannot establish causal relationships between exposure and outcomes; further, traditional observational studies may be affected by confounding factors and biases. Therefore, two-sample Mendelian randomization analysis was employed to enhance the robustness and credibility of the findings. The MR analysis utilized large-scale GWAS data, providing sufficient statistical power to assess the relationship between serum lipid metabolism and albuminuria. By combining cross-sectional study with MR analysis and employing the method of triangular validation, the close association between lipid metabolism and albuminuria has been explored from various perspectives, leading to more robust and reliable outcomes. This study also has limitations. This study referenced multiple studies that utilized the NHANES database to investigate the risk of albuminuria. We made efforts to identify factors associated with albuminuria and included as many covariates as possible, such as age, gender, race, education attainment, BMI, waist circumference, smoking, alcohol consumption, hypertension, and diabetes, to minimize potential confounding effects. Despite adjustments being made, unmeasured confounding factors may still impact the results. Some variables, such as diabetes status, were self-reported, which may introduce bias. Based on previous research, our definitions of the variables have received some support [29–31]. These pieces of evidence help mitigate potential biases and enhance the credibility of the research findings. Furthermore, combining self-reported data with clinical data can improve the overall reliability and validity of the study, which may be beneficial for future research. It is noteworthy that we observed a decreasing trend in serum LDL concentration as albuminuria occurrence and progression increased, contrary to traditional beliefs. This discrepancy may arise from potential interference of elevated oxidized LDL levels in NHANES when estimating LDL using the Friedewald formula, leading to underestimated values [74]. Further examination of these discrepancies is essential. Therefore, we plan to explore pertinent influencing factors, including dietary habits and interactions with other biomarkers, in our future research. Moreover, more precise measurement methods will reduce bias in exposure levels, leading to more robust conclusions. The presence of heterogeneity in the assessment of causal effects, as indicated by Cochran’s Q test in MR analysis, may arise from significant differences in sample characteristics—such as age, gender, and ethnicity—across the various studies. However, the random effects IVW MR is more effective in handling heterogeneity, ensuring a more accurate estimation of causal effects [75,76]. Additionally, we excluded SNPs with F-statistics below 10 to ensure the high quality and stability of the instrumental variables. Therefore, the results of the MR analysis remain robust. Additionally, due to the lack of GWAS data on albuminuria across different ethnic groups, the MR analysis was limited to European populations and cannot be generalized to other ethnic groups. Including data from diverse ethnic cohorts can enhance the study’s applicability. Due to the lack of data in the NHANES database, we could not conduct cross-sectional studies on more lipid metabolism markers (such as cholesterol esters in large HDL, cholesterol esters in large VLDL, cholesterol esters in medium LDL, cholesterol esters in medium VLDL, chylomicrons, and extremely large VLDL). To obtain more comprehensive and reliable conclusions, greater and more detailed database support is necessary. In our future research, we will focus on elucidating the potential associations between a broader range of lipid metabolism products and albuminuria. Additionally, we will explore the underlying mediators involved in these associations. By integrating cross-sectional studies with MR analysis, our aim is to provide new and comprehensive insights into the development and progression of kidney diseases.

## Conclusion

Combining observational studies with MR analysis results indicates a causal relationship between serum lipid metabolism and albuminuria risk. However, obtaining more comprehensive and reliable results necessitates larger databases providing a broader range of metabolic indicators. Further research is still required to elucidate the specific role of serum lipid metabolism in the occurrence and progression of future kidney and cardiovascular diseases.

## Supplementary Material

Supplementary Table Titles.docx

Figure S9.jpg

Figure S8.jpg

Figure S7.jpg

Table S2.xls

Table S1.xls

Table S5.docx

Supplementary Figure Legends.docx

Table S3.xls

Figure S3.jpg

Figure S5.jpg

Figure S1.jpg

Figure S4.jpg

Figure S6.jpg

Table S4.xls

Figure S10.jpg

Figure S2.jpg

## Data Availability

The datasets generated or analyzed during the current study are available in OpenGWAS database (https://gwas.mrcieu.ac.uk/). The dataset(s) supporting the conclusions of this article are available in the NHANES database: https://www.cdc.gov/nchs/nhanes/.
