# Supplementary material for: Lipid metabolism disorders and albuminuria risk: insights from National Health and Nutrition Examination Survey 2001–2018 and Mendelian randomization analyses
Source: Ren Fail. 2024 Nov 3;46(2):2420841. doi: 10.1080/0886022X.2024.2420841 (PMC11536668; doi:10.1080/0886022X.2024.2420841)
Supplement: Supplementary Table Titles.docx [file IRNF_A_2420841_SM2546.docx]

**Table S1.** Inclusion of instrumental variables

**Table S2.** MR analysis

**Table S3.** Sensitivity analysis

**Table S4.** Reverse MR analysis

**Table S5. MR-STROBE**
