# Supplementary figures and images for: Lipid metabolism disorders and albuminuria risk: insights from National Health and Nutrition Examination Survey 2001–2018 and Mendelian randomization analyses
Source: Ren Fail. 2024 Nov 3;46(2):2420841. doi: 10.1080/0886022X.2024.2420841 (PMC11536668; doi:10.1080/0886022X.2024.2420841)

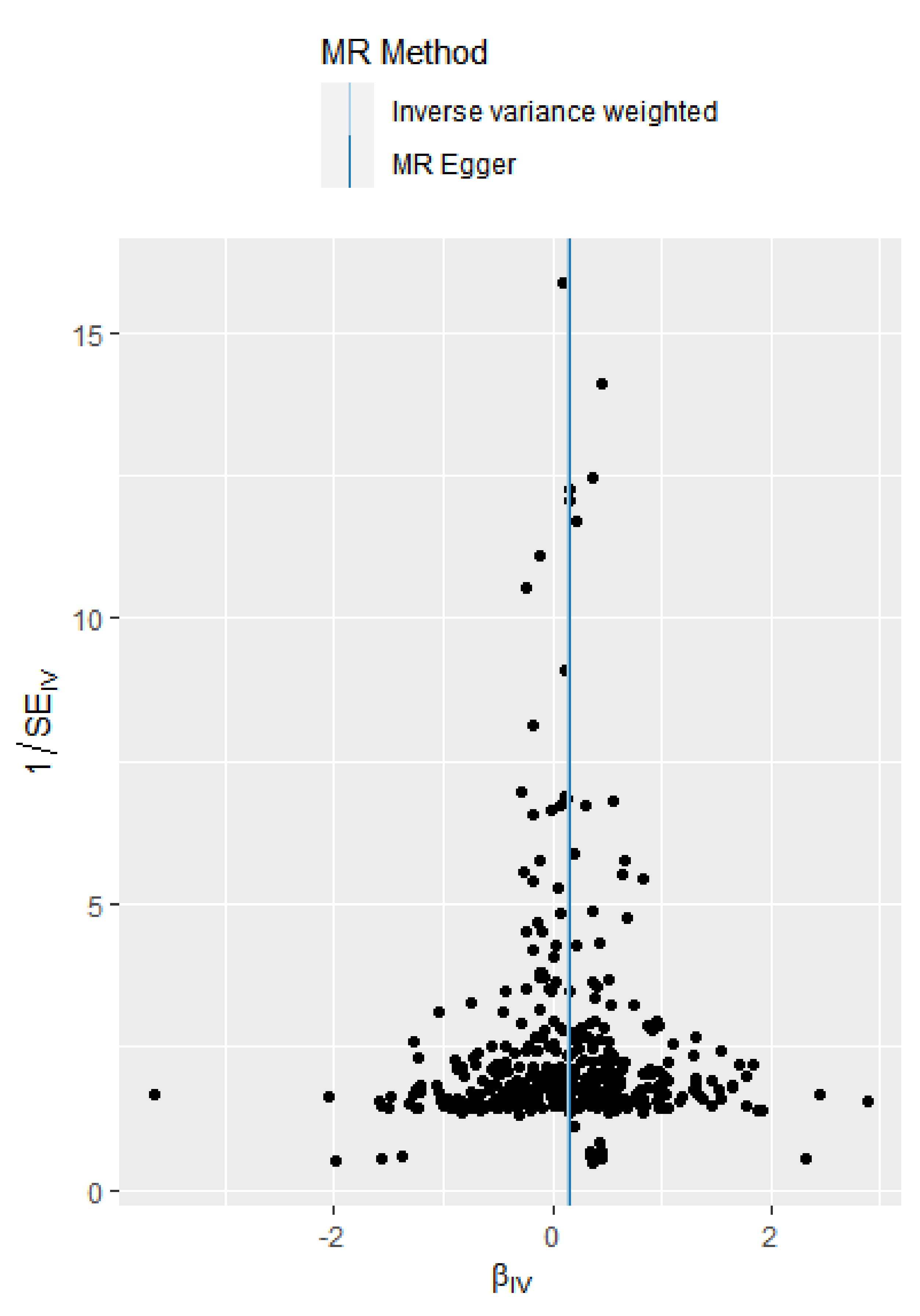

Supplement: Figure S8.jpg [file IRNF_A_2420841_SM2544.jpg]

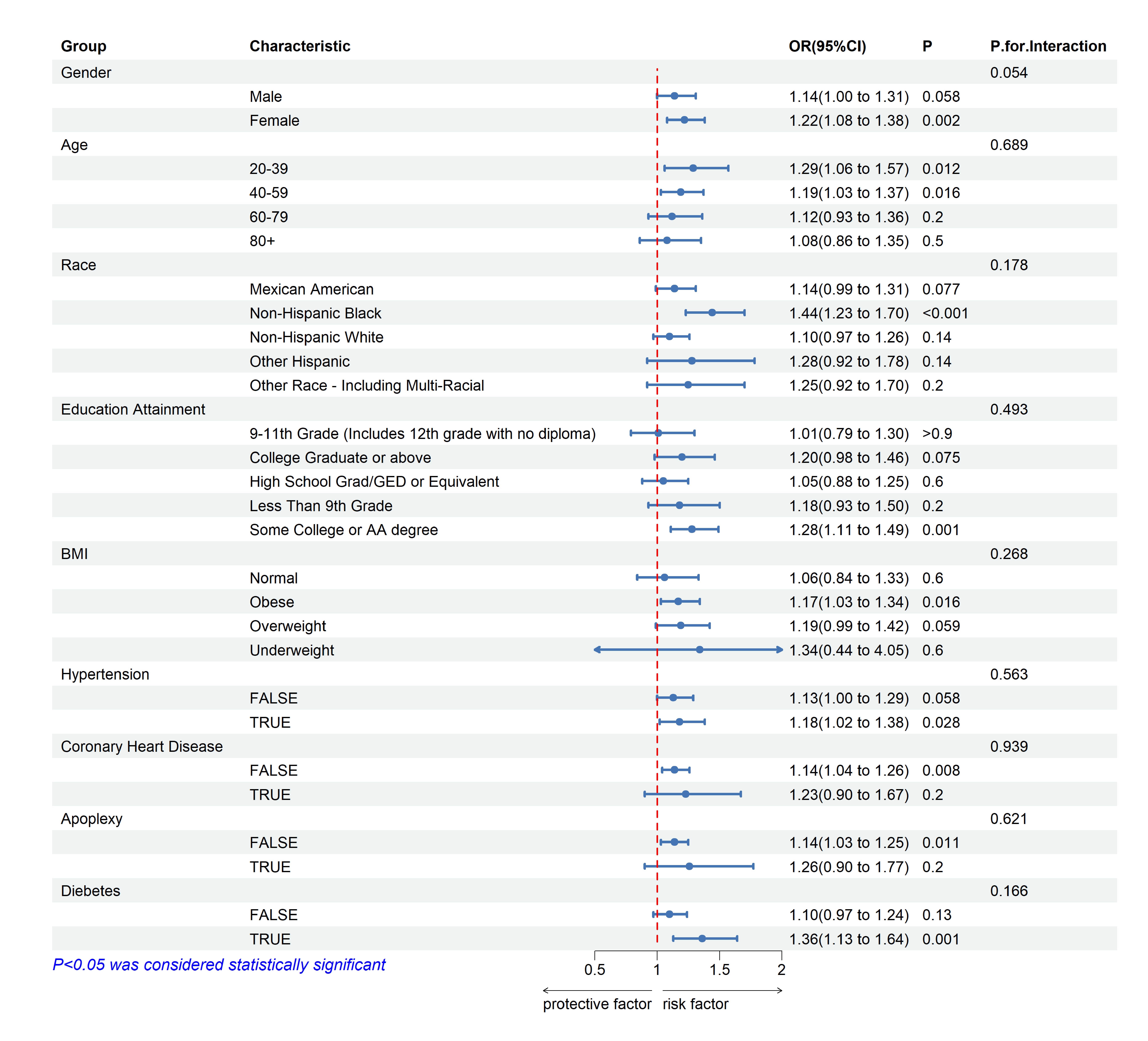

Supplement: Figure S1.jpg [file IRNF_A_2420841_SM2535.jpg]

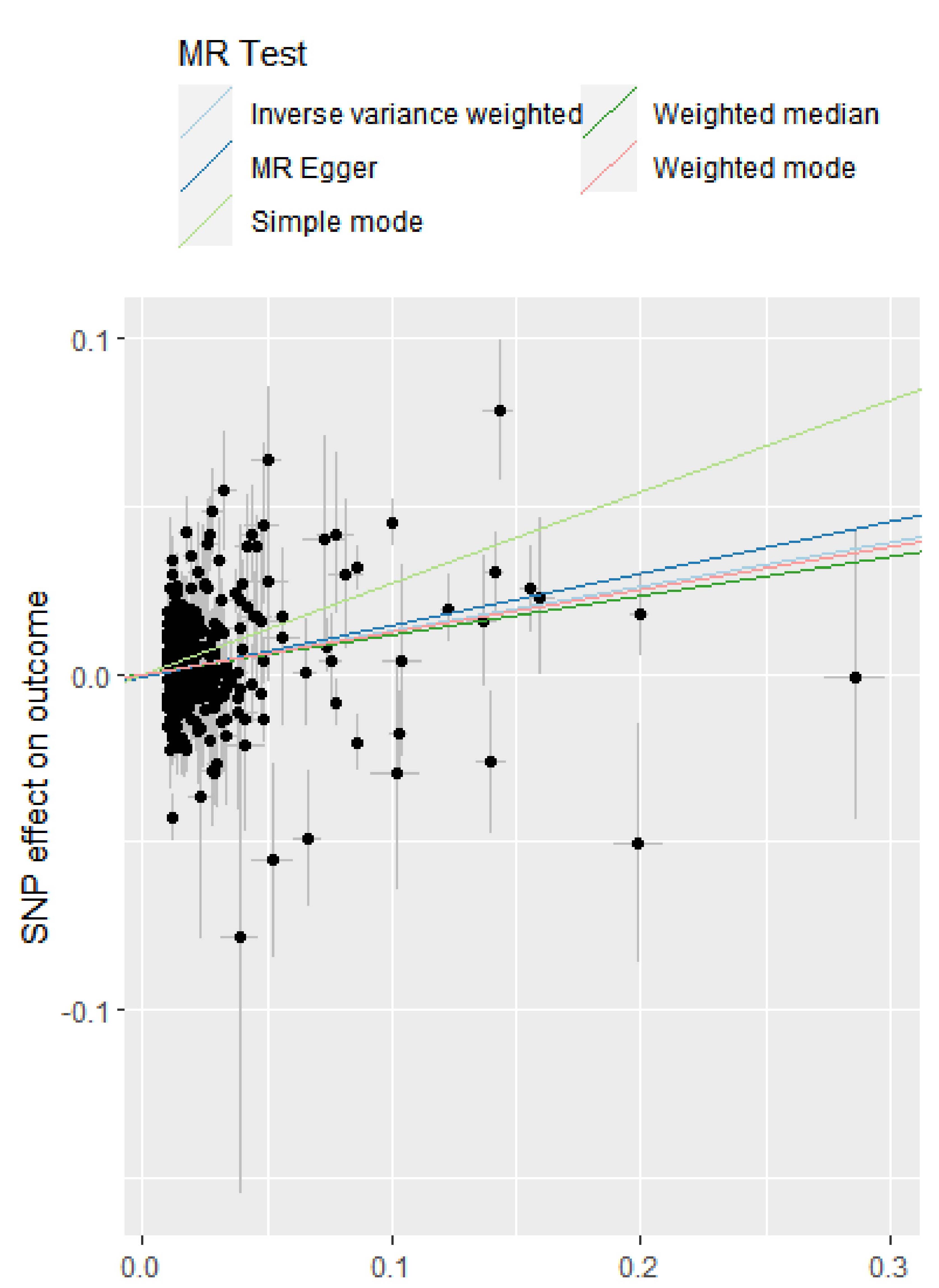

Supplement: Figure S6.jpg [file IRNF_A_2420841_SM2533.jpg]

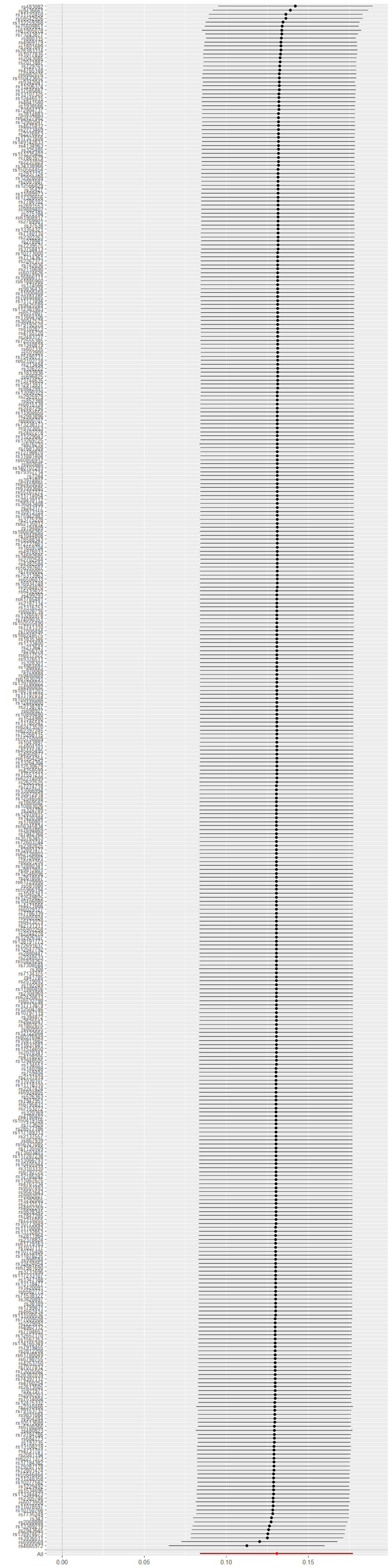

Supplement: Figure S10.jpg [file IRNF_A_2420841_SM2531.jpg]
