# Supplementary material for: Lipid metabolism disorders and albuminuria risk: insights from National Health and Nutrition Examination Survey 2001–2018 and Mendelian randomization analyses
Source: Ren Fail. 2024 Nov 3;46(2):2420841. doi: 10.1080/0886022X.2024.2420841 (PMC11536668; doi:10.1080/0886022X.2024.2420841)
Supplement: Table S5.docx [file IRNF_A_2420841_SM2540.docx]

**STROBE-MR checklist of recommended items to address in reports of Mendelian randomization studies**^1^ ^2^

| **Item No.** | **Section** | **Checklist item** | **Page No.** | **Relevant text from manuscript** |
| --- | --- | --- | --- | --- |
| 1 | **TITLE and ABSTRACT** | Indicate Mendelian randomization (MR) as the study’s design in the title and/or the abstract if that is a main purpose of the study | 1 | ‘Lipid Metabolism Disorders and Albuminuria Risk: Insights from National Health and Nutrition Examination Survey 2001–2018 and Mendelian Randomization Analyses’ The study incorporates MR analysis as a crucial component, as indicated in the title and abstract. |
|  | **INTRODUCTION** |  |  |  |
| 2 | **Background** | Explain the scientific background and rationale for the reported study. What is the exposure? Is a potential causal relationship between exposure and outcome plausible? Justify why MR is a helpful method to address the study question | 1, 2 | Increasing or decreasing levels of serum lipids cause various health effects in the human body, which are called disorders. These types of disorders usually increase triglyceride, low-density lipoprotein cholesterol (LDL), or both lipid levels. The body requires the useful fatty acid high-density lipoprotein cholesterol (HDL), which helps to transport bad cholesterol out of the body. Similarly, the accumulation of bad and unwanted lipids, such as fatty LDLs and triglyceride, damage the arteries and the kidney, and has serious consequences for cardiovascular and renal health (Natesan and Kim, 2021). Recently, Xiao et al. (2021) published an article on inherited complex lipid metabolism disorders, stating that over 80 diseases have been identified as complex lipid metabolism defects (Xiao et al., 2021) with main consequences of chronic kidney disease (CKD) and a higher incidence of cardiovascular events (Soppert et al., 2020; Baek et al., 2022). For instance, fluctuations in levels of certain lipid metabolites, such as triglycerides and decreased HDL, may be associated with renal impairment (Chen et al., 2017; Lin et al., 2022). Additionally, disrupted cholesterol balance in the body may induce the development of diabetes and exacerbate the risk of albuminuria (Moriwaki et al., 2003; Chen et al., 2021b). Reduced HDL levels and elevated LDL and triglycerides are also significant risk factors for various cardiovascular diseases, such as coronary heart disease and apoplexy (Kale et al., 2023). Moreover, dyslipidemia is a significant complication of CKD, and in severe renal failure patients, it may even lead to a further decreased levels of LDL and cholesterol (Vaziri, 2006). The consequences of lipid metabolism disorders are complex. Identifying commonalities among them may be the key to preventing and solving these diseases. At this point, albuminuria appeared in the researchers' field of view. Albuminuria, defined as a urinary albumin-to-creatinine ratio (UACR) greater than 30 mg/g, is often seen in patients with lipid metabolism disorders (Bobulescu, 2010; Sun et al., 2015). Within this range, a UACR of 30-300 mg/g is defined as microalbuminuria, while a UACR greater than 300 mg/g is defined as macroalbuminuria (Boorsma et al., 2023). The researchers found that microalbuminuria can serve as an indicator of renal function impairment and a sensitive marker for the early progression of chronic kidney disease (Futrakul et al., 2009). Further, the degree of albuminuria is closely related to the prognosis of renal conditions, elevated levels of albuminuria frequently indicate worsening of the disease and unfavorable results (Khanijou et al., 2022). Meanwhile, albuminuria is also a potential biomarker for various cardiovascular diseases (Barzilay et al., 2024). There is evidence indicating that albuminuria increases the risk of apoplexy and coronary heart disease (Barzilay et al., 2024). It is worth noting that patients with lipid metabolism disorders may face an increased risk of albuminuria (Tian et al., 2023). Obviously, albuminuria serves as a significant biomarker for cardiorenal diseases and exhibits intricate associations with lipid metabolism disorders. Additionally, the role of serum lipid metabolism levels in screening and treating albuminuria should also be taken into consideration. However, the causal relationship and specific mechanisms between lipid metabolism and albuminuria are not fully understood, and there is a lack of detailed research specifically linking the two fields. |
| 3 | **Objectives** | State specific objectives clearly, including pre-specified causal hypotheses (if any). State that MR is a method that, under specific assumptions, intends to estimate causal effects | 2, 4 | Genetic instrumental variables were selected from various genome-wide association studies (GWAS) to validate the causal relationship between exposure and outcome. In addition to serum levels of total triglycerides, cholesterol, HDL, and LDL, we also included more exposure indicators that can represent the level of serum lipid metabolism, such as cholesterol esters in large HDL, cholesterol esters in large VLDL, cholesterol esters in medium HDL, cholesterol esters in medium LDL, cholesterol esters in medium VLDL, chylomicrons, and extremely large VLDL particles (Kettunen et al., 2016; Richardson et al., 2020; Richardson et al., 2022). The genetic variants related to serum lipid metabolism in these GWAS studies are derived from European populations. In this study, the relationship between serum lipid metabolism and albuminuria was investigated by stratifying serum triglycerides, cholesterol, HDL, and LDL concentrations of participants in the National Health and Nutrition Examination Survey (NHANES). Two-sample Mendelian randomization (MR) was employed by using single-nucleotide polymorphisms (SNPs) from genome-wide association studies as instrumental variables (IVs) to simulate a randomized controlled trial (RCT) and investigate the unidirectional causal relationship between exposure and outcome (Birney, 2022). In summary, we aim to investigate the causal relationship between lipid metabolism disorders and the risk of albuminuria from both a population and genetic perspective. |
|  | **METHODS** |  |  |  |
| 4 | **Study design and data sources** | Present key elements of the study design early in the article. Consider including a table listing sources of data for all phases of the study. For each data source contributing to the analysis, describe the following: | 2, 4, 5 | Two-sample Mendelian randomization (MR) was employed by using single-nucleotide polymorphisms (SNPs) from genome-wide association studies as instrumental variables (IVs) to simulate a randomized controlled trial (RCT) and investigate the unidirectional causal relationship between exposure and outcome (Birney, 2022). In summary, we aim to investigate the causal relationship between lipid metabolism disorders and the risk of albuminuria from both a population and genetic perspective.  Genetic instrumental variables were selected from various genome-wide association studies (GWAS) to validate the causal relationship between exposure and outcome. In addition to serum levels of total triglycerides, cholesterol, HDL, and LDL, we also included more exposure indicators that can represent the level of serum lipid metabolism, such as cholesterol esters in large HDL, cholesterol esters in large VLDL, cholesterol esters in medium HDL, cholesterol esters in medium LDL, cholesterol esters in medium VLDL, chylomicrons, and extremely large VLDL particles (Kettunen et al., 2016; Richardson et al., 2020; Richardson et al., 2022). The genetic variants related to serum lipid metabolism in these GWAS studies are derived from European populations. The GWAS summary data on albuminuria is based on a large meta-analysis (n=348,954, including 51,861 cases and 297,093 controls), with detailed information available on CKDgen (<https://ckdgen.imbi.uni-freiburg.de/datasets>) (Teumer et al., 2019).  Standardized quality assessment was employed to screen instrumental variables, ensuring the rigor and reliability of MR analysis. In order to improve the thoroughness of the results, a lenient genome-wide significance cutoff of P < 5×10-6 for the selection of SNPs linked to lipid metabolism exposure traits was utilized. An evaluation of Linkage Disequilibrium (LD) to ensure the independence of the chosen instrumental variables (IVs), maintaining an LD r2 threshold of less than 0.001 within a 10 MB range, was carried out. The computation of the F-statistic was performed for each SNP, and those with F values below 10 were excluded to mitigate any potential bias arising from weak instruments. Throughout the standardization procedure, SNPs that were incompatible or displayed a palindromic structure with intermediate allele frequency were omitted (Burgess et al., 2019; Huang et al., 2023; Larsson et al., 2023). In the Reverse-MR analysis, albuminuria was considered as the exposure factor and serum lipid metabolism indicators as the outcome, repeating the aforementioned process. Figure 2 illustrates the flowchart of MR and the three core assumptions of MR.  In MR analysis, we consider the IVW method results as the primary analytical outcomes. Additionally, these results were validated by using MR-Egger, weighted median, simple mode, and weighted mode methods. In case of contradictory findings, the IVW results remain unaffected. Detailed descriptions of these methods have been provided in previous studies (Bowden and Holmes, 2019; Chen et al., 2021a). Cochran’s Q test was employed to assess the outcomes of IVW and MR-Egger analyses (P<0.05 indicates heterogeneity). The MR-Egger intercept test was employed to examine the horizontal pleiotropy of SNPs, where the regression intercept assesses the magnitude of horizontal pleiotropy (P<0.05 indicates potential pleiotropy). Additionally, leave-one-out analysis and funnel plots were conducted for heterogeneity assessment (Chen et al., 2021a). |
|  | a) | Setting: Describe the study design and the underlying population, if possible. Describe the setting, locations, and relevant dates, including periods of recruitment, exposure, follow-up, and data collection, when available. | 4, 6 | The genetic variants related to serum lipid metabolism in these GWAS studies are derived from European populations. The GWAS summary data on albuminuria is based on a large meta-analysis (n=348,954, including 51,861 cases and 297,093 controls), with detailed information available on CKDgen (<https://ckdgen.imbi.uni-freiburg.de/datasets>) (Teumer et al., 2019). We identified a series of SNPs related to serum lipid metabolism (Cholesterol esters in large HDL, Cholesterol esters in large VLDL, Cholesterol esters in medium HDL, Cholesterol esters in medium LDL, Cholesterol esters in medium VLDL, chylomicrons, and extremely large VLDL particles). Detailed information provided in Supplementary Table S3. |
|  | b) | Participants: Give the eligibility criteria, and the sources and methods of selection of participants. Report the sample size, and whether any power or sample size calculations were carried out prior to the main analysis | 4, 6 | The genetic variants related to serum lipid metabolism in these GWAS studies are derived from European populations. The GWAS summary data on albuminuria is based on a large meta-analysis (n=348,954, including 51,861 cases and 297,093 controls), with detailed information available on CKDgen (<https://ckdgen.imbi.uni-freiburg.de/datasets>) (Teumer et al., 2019). We identified a series of SNPs related to serum lipid metabolism (Cholesterol esters in large HDL, Cholesterol esters in large VLDL, Cholesterol esters in medium HDL, Cholesterol esters in medium LDL, Cholesterol esters in medium VLDL, chylomicrons, and extremely large VLDL particles). Detailed information provided in Supplementary Table S3. |
|  | c) | Describe measurement, quality control and selection of genetic variants | 4 | Standardized quality assessment was employed to screen instrumental variables, ensuring the rigor and reliability of MR analysis. In order to improve the thoroughness of the results, a lenient genome-wide significance cutoff of P < 5×10-6 for the selection of SNPs linked to lipid metabolism exposure traits was utilized. An evaluation of Linkage Disequilibrium (LD) to ensure the independence of the chosen instrumental variables (IVs), maintaining an LD r2 threshold of less than 0.001 within a 10 MB range, was carried out. The computation of the F-statistic was performed for each SNP, and those with F values below 10 were excluded to mitigate any potential bias arising from weak instruments. Throughout the standardization procedure, SNPs that were incompatible or displayed a palindromic structure with intermediate allele frequency were omitted (Burgess et al., 2019; Huang et al., 2023; Larsson et al., 2023). In the Reverse-MR analysis, albuminuria was considered as the exposure factor and serum lipid metabolism indicators as the outcome, repeating the aforementioned process. Figure 2 illustrates the flowchart of MR and the three core assumptions of MR. |
|  | d) | For each exposure, outcome, and other relevant variables, describe methods of assessment and diagnostic criteria for diseases | 4 | Genetic instrumental variables were selected from various genome-wide association studies (GWAS) to validate the causal relationship between exposure and outcome. In addition to serum levels of total triglycerides, cholesterol, HDL, and LDL, we also included more exposure indicators that can represent the level of serum lipid metabolism, such as cholesterol esters in large HDL, cholesterol esters in large VLDL, cholesterol esters in medium HDL, cholesterol esters in medium LDL, cholesterol esters in medium VLDL, chylomicrons, and extremely large VLDL particles (Kettunen et al., 2016; Richardson et al., 2020; Richardson et al., 2022). The genetic variants related to serum lipid metabolism in these GWAS studies are derived from European populations. The GWAS summary data on albuminuria is based on a large meta-analysis (n=348,954, including 51,861 cases and 297,093 controls), with detailed information available on CKDgen (<https://ckdgen.imbi.uni-freiburg.de/datasets>) (Teumer et al., 2019). The definitions of serum lipid are derived from corresponding traits in the GWAS catalog. Albuminuria was defined as a UACR of '> 30 mg/g'. |
|  | e) | Provide details of ethics committee approval and participant informed consent, if relevant | 9 | The studies involving human participants were granted ethical approval by the NCHS Research Ethics Review Board. These studies were carried out in compliance with local legislation and institutional requirements. Prior to participation in this study, all participants provided written informed consent. Our research was conducted using publicly available anonymized databases, namely GWAS and FinnGen, which are exempt from ethical compliance. |
| 5 | **Assumptions** | Explicitly state the three core IV assumptions for the main analysis (relevance, independence and exclusion restriction) as well assumptions for any additional or sensitivity analysis |  | Standardized quality assessment was employed to screen instrumental variables, ensuring the rigor and reliability of MR analysis. In order to improve the thoroughness of the results, a lenient genome-wide significance cutoff of P < 5×10-6 for the selection of SNPs linked to lipid metabolism exposure traits was utilized. An evaluation of Linkage Disequilibrium (LD) to ensure the independence of the chosen instrumental variables (IVs), maintaining an LD r2 threshold of less than 0.001 within a 10 MB range, was carried out. The computation of the F-statistic was performed for each SNP, and those with F values below 10 were excluded to mitigate any potential bias arising from weak instruments. Throughout the standardization procedure, SNPs that were incompatible or displayed a palindromic structure with intermediate allele frequency were omitted (Burgess et al., 2019; Huang et al., 2023; Larsson et al., 2023). In the Reverse-MR analysis, albuminuria was considered as the exposure factor and serum lipid metabolism indicators as the outcome, repeating the aforementioned process. Figure 2 illustrates the flowchart of MR and the three core assumptions of MR.  Figure 2. Study Design in MR: Assumption Ⅰ: IVs was strongly correlated with exposure. Assumption Ⅱ: IVs was not associated with confounders. Assumption Ⅲ: IVs was not associated with outcome. |
| 6 | **Statistical methods: main analysis** | Describe statistical methods and statistics used |  |  |
|  | a) | Describe how quantitative variables were handled in the analyses (i.e., scale, units, model) | 5 | In MR analysis, we consider the IVW method results as the primary analytical outcomes. Additionally, these results were validated by using MR-Egger, weighted median, simple mode, and weighted mode methods. In case of contradictory findings, the IVW results remain unaffected. Detailed descriptions of these methods have been provided in previous studies (Bowden and Holmes, 2019; Chen et al., 2021a). |
|  | b) | Describe how genetic variants were handled in the analyses and, if applicable, how their weights were selected | 4 | Standardized quality assessment was employed to screen instrumental variables, ensuring the rigor and reliability of MR analysis. In order to improve the thoroughness of the results, a lenient genome-wide significance cutoff of P < 5×10-6 for the selection of SNPs linked to lipid metabolism exposure traits was utilized. An evaluation of Linkage Disequilibrium (LD) to ensure the independence of the chosen instrumental variables (IVs), maintaining an LD r2 threshold of less than 0.001 within a 10 MB range, was carried out. The computation of the F-statistic was performed for each SNP, and those with F values below 10 were excluded to mitigate any potential bias arising from weak instruments. Throughout the standardization procedure, SNPs that were incompatible or displayed a palindromic structure with intermediate allele frequency were omitted (Burgess et al., 2019; Huang et al., 2023; Larsson et al., 2023). In the Reverse-MR analysis, albuminuria was considered as the exposure factor and serum lipid metabolism indicators as the outcome, repeating the aforementioned process. Figure 2 illustrates the flowchart of MR and the three core assumptions of MR. |
|  | c) | Describe the MR estimator (e.g. two-stage least squares, Wald ratio) and related statistics. Detail the included covariates and, in case of two-sample MR, whether the same covariate set was used for adjustment in the two samples |  | Not applicable. |
|  | d) | Explain how missing data were addressed |  | Not applicable. |
|  | e) | If applicable, indicate how multiple testing was addressed |  | Not applicable. |
| 7 | **Assessment of assumptions** | Describe any methods or prior knowledge used to assess the assumptions or justify their validity | 4 | The computation of the F-statistic was performed for each SNP, and those with F values below 10 were excluded to mitigate any potential bias arising from weak instruments. |
| 8 | **Sensitivity analyses and additional analyses** | Describe any sensitivity analyses or additional analyses performed (e.g. comparison of effect estimates from different approaches, independent replication, bias analytic techniques, validation of instruments, simulations) | 4, 5 | The computation of the F-statistic was performed for each SNP, and those with F values below 10 were excluded to mitigate any potential bias arising from weak instruments. Cochran’s Q test was employed to assess the outcomes of IVW and MR-Egger analyses (P<0.05 indicates heterogeneity). The MR-Egger intercept test was employed to examine the horizontal pleiotropy of SNPs, where the regression intercept assesses the magnitude of horizontal pleiotropy (P<0.05 indicates potential pleiotropy). Additionally, leave-one-out analysis and funnel plots were conducted for heterogeneity assessment (Chen et al., 2021a). |
| 9 | **Software and pre-registration** |  |  |  |
|  | a) | Name statistical software and package(s), including version and settings used |  | The “TwoSampleMR” package (version 0.5.7) in R (version 4.3.1) was utilized for MR analysis. |
|  | b) | State whether the study protocol and details were pre-registered (as well as when and where) |  | Not registered. |
|  | **RESULTS** |  |  |  |
| 10 | **Descriptive data** |  |  |  |
|  | a) | Report the numbers of individuals at each stage of included studies and reasons for exclusion. Consider use of a flow diagram | 5 | In MR analysis, we consider the IVW method results as the primary analytical outcomes. Additionally, these results were validated by using MR-Egger, weighted median, simple mode, and weighted mode methods. The analysis did not include adjustments for age, gender, and study location. |
|  | b) | Report summary statistics for phenotypic exposure(s), outcome(s), and other relevant variables (e.g. means, SDs, proportions) |  | Not applicable |
|  | c) | If the data sources include meta-analyses of previous studies, provide the assessments of heterogeneity across these studies |  | Not applicable |
|  | d) | For two-sample MR:  i.  Provide justification of the similarity of the genetic variant-exposure associations between the exposure and outcome samples  ii.  Provide information on the number of individuals who overlap between the exposure and outcome studies | 4 | The genetic variants related to serum lipid metabolism in these GWAS studies are derived from European populations. The GWAS summary data on albuminuria is based on a large meta-analysis (n=348,954, including 51,861 cases and 297,093 controls), with detailed information available on CKDgen (<https://ckdgen.imbi.uni-freiburg.de/datasets>) (Teumer et al., 2019). |
| 11 | **Main results** |  |  |  |
|  | a) | Report the associations between genetic variant and exposure, and between genetic variant and outcome, preferably on an interpretable scale | 6 | We identified a series of SNPs related to serum lipid metabolism (Cholesterol esters in large HDL, Cholesterol esters in large VLDL, Cholesterol esters in medium HDL, Cholesterol esters in medium LDL, Cholesterol esters in medium VLDL, chylomicrons, and extremely large VLDL particles). Detailed information provided in Supplementary Table S3. |
|  | b) | Report MR estimates of the relationship between exposure and outcome, and the measures of uncertainty from the MR analysis, on an interpretable scale, such as odds ratio or relative risk per SD difference | 6 | The MR analysis results indicate that Cholesterol esters in large HDL (IVW: OR = 0.95, 95% CI = 0.92-0.98, P = 0.003) and Serum total HDL level (IVW: OR = 0.91, 95% CI = 0.86-0.97, P = 0.002) are potential protective factors for MA. Conversely, Cholesterol esters in large VLDL (IVW: OR = 1.07, 95% CI = 1.04-1.10, P < 0.001), Cholesterol esters in medium LDL (IVW: OR = 1.06, 95% CI = 1.03-1.09, P < 0.001), Cholesterol esters in medium VLDL (IVW: OR = 1.05, 95% CI = 1.00-1.10, P = 0.032), Chylomicrons and extremely large VLDL particles (IVW: OR = 1.08, 95% CI = 1.03-1.14, P = 0.003), and Triglycerides (IVW: OR = 1.14, 95% CI = 1.09-1.19, P < 0.001) pose risks for albuminuria. No causal relationship was found between Serum total LDL level, Serum Total cholesterol level, and albuminuria (Figure 5). The findings are further supported by scatter plots, providing additional evidence. Detailed information provided in Supplementary Figure S5, S6 and Supplementary Table S4. |
|  | c) | If relevant, consider translating estimates of relative risk into absolute risk for a meaningful time period | 6 | The findings revealed that several lipid markers are positively associated with albuminuria risk. Specifically, elevated serum triglyceride concentration, cholesterol esters in large VLDL particles, extremely large VLDL particles, cholesterol esters in medium LDL particles, and cholesterol esters in medium VLDL particles showed a positive correlation with albuminuria risk. On the other hand, it was observed that serum HDL concentration, including cholesterol esters in large HDL particles, displayed a potential protective effect against albuminuria. However, no significant correlation was found between serum total cholesterol or LDL concentrations and albuminuria. |
|  | d) | Consider plots to visualize results (e.g. forest plot, scatterplot of associations between genetic variants and outcome versus between genetic variants and exposure) | 6 | The findings are further supported by scatter plots, providing additional evidence. Detailed information provided in Supplementary Figure S5, S6 and Supplementary Table S4. Funnel plots and leave-one-out plots further validate the above findings. Detailed information provided in Supplementary Figure S7, S8, S9 and S10. |
| 12 | **Assessment of assumptions** |  |  |  |
|  | a) | Report the assessment of the validity of the assumptions | 6 | We identified a series of SNPs related to serum lipid metabolism (Cholesterol esters in large HDL, Cholesterol esters in large VLDL, Cholesterol esters in medium HDL, Cholesterol esters in medium LDL, Cholesterol esters in medium VLDL, chylomicrons, and extremely large VLDL particles). Detailed information provided in Supplementary Table S3. The MR analysis results indicate that Cholesterol esters in large HDL (IVW: OR = 0.95, 95% CI = 0.92-0.98, P = 0.003) and Serum total HDL level (IVW: OR = 0.91, 95% CI = 0.86-0.97, P = 0.002) are potential protective factors for MA. Conversely, Cholesterol esters in large VLDL (IVW: OR = 1.07, 95% CI = 1.04-1.10, P < 0.001), Cholesterol esters in medium LDL (IVW: OR = 1.06, 95% CI = 1.03-1.09, P < 0.001), Cholesterol esters in medium VLDL (IVW: OR = 1.05, 95% CI = 1.00-1.10, P = 0.032), Chylomicrons and extremely large VLDL particles (IVW: OR = 1.08, 95% CI = 1.03-1.14, P = 0.003), and Triglycerides (IVW: OR = 1.14, 95% CI = 1.09-1.19, P < 0.001) pose risks for albuminuria. No causal relationship was found between Serum total LDL level, Serum Total cholesterol level, and albuminuria (Figure 5). The findings are further supported by scatter plots, providing additional evidence. Detailed information provided in Supplementary Figure S5, S6 and Supplementary Table S4. Further, some exposure indicators analyzed in this study exhibited heterogeneity, yet directional pleiotropy was not present, thus not affecting the stability of the IVW results (Table S5). Funnel plots and leave-one-out plots further validate the above findings. Detailed information provided in Supplementary Figure S7, S8, S9 and S10. In the reverse Mendelian randomization analysis, we did not identify an association between MA and the included serum metabolic markers, demonstrating a unidirectional causal relationship between exposure and outcome. Detailed information provided in Supplementary Table S6. |
|  | b) | Report any additional statistics (e.g., assessments of heterogeneity across genetic variants, such as *I^2^*, Q statistic or E-value) |  | Not applicable |
| 13 | **Sensitivity analyses and additional analyses** |  |  |  |
|  | a) | Report any sensitivity analyses to assess the robustness of the main results to violations of the assumptions | 6 | The MR analysis results indicate that Cholesterol esters in large HDL (IVW: OR = 0.95, 95% CI = 0.92-0.98, P = 0.003) and Serum total HDL level (IVW: OR = 0.91, 95% CI = 0.86-0.97, P = 0.002) are potential protective factors for MA. Conversely, Cholesterol esters in large VLDL (IVW: OR = 1.07, 95% CI = 1.04-1.10, P < 0.001), Cholesterol esters in medium LDL (IVW: OR = 1.06, 95% CI = 1.03-1.09, P < 0.001), Cholesterol esters in medium VLDL (IVW: OR = 1.05, 95% CI = 1.00-1.10, P = 0.032), Chylomicrons and extremely large VLDL particles (IVW: OR = 1.08, 95% CI = 1.03-1.14, P = 0.003), and Triglycerides (IVW: OR = 1.14, 95% CI = 1.09-1.19, P < 0.001) pose risks for albuminuria. No causal relationship was found between Serum total LDL level, Serum Total cholesterol level, and albuminuria (Figure 5). The findings are further supported by scatter plots, providing additional evidence. Detailed information provided in Supplementary Figure S5, S6 and Supplementary Table S4.  Further, some exposure indicators analyzed in this study exhibited heterogeneity, yet directional pleiotropy was not present, thus not affecting the stability of the IVW results (Table S5). Funnel plots and leave-one-out plots further validate the above findings. Detailed information provided in Supplementary Figure S7, S8, S9 and S10. |
|  | b) | Report results from other sensitivity analyses or additional analyses | 6 | Funnel plots and leave-one-out plots further validate the above findings. Detailed information provided in Supplementary Figure S7, S8, S9 and S10. |
|  | c) | Report any assessment of direction of causal relationship (e.g., bidirectional MR) | 6 | In the reverse Mendelian randomization analysis, we did not identify an association between MA and the included serum metabolic markers, demonstrating a unidirectional causal relationship between exposure and outcome. Detailed information provided in Supplementary Table S6. |
|  | d) | When relevant, report and compare with estimates from non-MR analyses | 6 | In this study, a cross-sectional analysis was conducted using data from the nationally representative NHANES 2001-2018 in the United States. Two-sample MR was employed to examine the potential causal relationship between lipid metabolism and the risk of albuminuria. The findings revealed that several lipid markers are positively associated with albuminuria risk. Specifically, elevated serum triglyceride concentration, cholesterol esters in large VLDL particles, extremely large VLDL particles, cholesterol esters in medium LDL particles, and cholesterol esters in medium VLDL particles showed a positive correlation with albuminuria risk. On the other hand, it was observed that serum HDL concentration, including cholesterol esters in large HDL particles, displayed a potential protective effect against albuminuria. However, no significant correlation was found between serum total cholesterol or LDL concentrations and albuminuria. |
|  | e) | Consider additional plots to visualize results (e.g., leave-one-out analyses) | 6 | Detailed information provided in Supplementary Figure S7, S8, S9 and S10. |
|  | **DISCUSSION** |  |  |  |
| 14 | **Key results** | Summarize key results with reference to study objectives | 6 | Specifically, elevated serum triglyceride concentration, cholesterol esters in large VLDL particles, extremely large VLDL particles, cholesterol esters in medium LDL particles, and cholesterol esters in medium VLDL particles showed a positive correlation with albuminuria risk. On the other hand, it was observed that serum HDL concentration, including cholesterol esters in large HDL particles, displayed a potential protective effect against albuminuria. However, no significant correlation was found between serum total cholesterol or LDL concentrations and albuminuria. |
| 15 | **Limitations** | Discuss limitations of the study, taking into account the validity of the IV assumptions, other sources of potential bias, and imprecision. Discuss both direction and magnitude of any potential bias and any efforts to address them |  |  |
| 16 | **Interpretation** |  |  |  |
|  | a) | Meaning: Give a cautious overall interpretation of results in the context of their limitations and in comparison with other studies | 6, 7, 8 | The relationship between lipid metabolism and albuminuria has long been a subject of considerable interest. Some researchers suggest a close association between the triglyceride-glucose (Ty-G) index and urinary albumin concentration (Wang et al., 2023). Evidence suggests a potential association between the urinary albumin-creatinine ratio and visceral obesity index (Li et al., 2022a). A cohort study revealed that higher serum triglyceride levels are associated with a decline in renal glomerular filtration rate (GFR) and an increased incidence of cardiovascular diseases, accompanied by albuminuria (Cui et al., 2023). Lipid metabolites play a crucial role as important cellular signaling molecules in the pathogenesis and progression of cardiorenal diseases. Lipid metabolism abnormalities commonly lead to lipid accumulation and metabolic dysfunction in renal diseases, subsequently triggering pathological processes such as oxidative stress, inflammation, and tissue damage (Bugarski et al., 2021; Chen et al., 2022; Li et al., 2022b). For instance, elevated levels of triglycerides, an important lipid metabolite, are closely associated with renal dysfunction, glomerular injury, and increased albuminuria in renal diseases (Pauley et al., 2023). Further, abnormal triglyceride accumulation can contribute to oxidative stress and inflammatory responses within the kidneys (Au-Yeung et al., 2023). Elevated levels of triglycerides are also associated with an increased risk of cardiovascular diseases, such as atherosclerosis and coronary heart disease (Chait, 2022). In this study, we found a positive correlation between triglyceride levels and albuminuria, regardless of whether triglycerides were examined as continuous or outcome variables. Moreover, MR analysis provided robust evidence at the genetic level, confirming and strengthening the causal relationship between triglycerides and albuminuria.  In addition, VLDL and LDL may have adverse effects in kidney diseases. Elevated levels of VLDL and LDL are associated with glomerular lesions, interstitial fibrosis, and tubular cell damage, which can intensify the progression of kidney diseases and lead to albumin leakage into the urine (Wahl et al., 2016; Huang and Lee, 2022). Moreover, increased levels of VLDL and LDL may induce and aggravate renal inflammation, activating inflammatory cells and promoting immune cell migration (Wahl et al., 2016). These lipoproteins are also associated with an increased risk of atherosclerosis and coronary heart disease (Akers et al., 2019). In this study, we observed a significant causal relationship only between cholesterol esters in medium LDL metabolites and albuminuria. Additionally, we found that certain metabolites associated with a series of VLDL (cholesterol esters in large VLDL particles, extremely large VLDL particles, and cholesterol esters in medium VLDL particles) were related to the risk of albuminuria. The findings provide detailed insights into the complex correlations between specific lipid metabolites and albuminuria.  HDL, with its reverse cholesterol transport function, facilitates the clearance of cholesterol from arterial walls and its transport to the liver for metabolism, thus lowering the risk of atherosclerosis and coronary heart disease (Ossoli et al., 2019). However, the role of HDL in kidney diseases is more complex. While HDL is generally considered to have a protective effect on the glomerular filtration barrier, in specific kidney diseases, elevated HDL levels may be associated with increased oxidative stress and inflammation in the glomerular basement membrane, leading to albumin leakage (Zhong et al., 2019). Several studies have suggested that higher HDL levels may be correlated with the activation of inflammatory cells and increased inflammation in kidney diseases (Strazzella et al., 2021). In contrast, MR analysis has provided evidence of a negative correlation between HDL and the risk of albuminuria, further highlighting the causal relationship between HDL and albuminuria.  Some researchers argue that serum total cholesterol is a significant risk factor for albuminuria, however, at the genetic level, our view is different, as we found no significant association between serum total cholesterol and the risk of albuminuria (Nam et al., 2015). we presume this may be attributed to the fact that they restricted their study population to individuals in the prediabetic stage.  It is noteworthy that there is an interplay between genetic and environmental factors in the relationship between lipid metabolism and albuminuria (Cerdena et al., 2021). In this study, we found differences in the risk of albuminuria associated with lipid metabolism disorders among different racial subgroups, particularly in Non-Hispanic Black individuals who exhibited an increased risk of developing the condition. Further, factors such as gender, age, education level, BMI, and the presence of comorbidities (e.g., hypertension, coronary heart disease, stroke, diabetes) also influence the individual's susceptibility to albuminuria risk in the context of lipid metabolism disorders. In clinical management, maintaining a healthy lipid metabolism may help prevent or alleviate the occurrence of microalbuminuria and related kidney diseases (Reidy and Ross, 2021). Given the differences in lipid metabolism among individual patients, personalized therapy plays a crucial role in clinical management. We suggest that, for patients at high risk of abnormal lipid metabolism and microalbuminuria, doctors should tailor specific treatment plans based on their unique conditions, incorporating lipid regulation, renal protection, and other measures to achieve more effective therapeutic outcomes. |
|  | b) | Mechanism: Discuss underlying biological mechanisms that could drive a potential causal relationship between the investigated exposure and the outcome, and whether the gene-environment equivalence assumption is reasonable. Use causal language carefully, clarifying that IV estimates may provide causal effects only under certain assumptions | 6, 7, 8 | Lipid metabolism abnormalities commonly lead to lipid accumulation and metabolic dysfunction in renal diseases, subsequently triggering pathological processes such as oxidative stress, inflammation, and tissue damage (Bugarski et al., 2021; Chen et al., 2022; Li et al., 2022b). For instance, elevated levels of triglycerides, an important lipid metabolite, are closely associated with renal dysfunction, glomerular injury, and increased albuminuria in renal diseases (Pauley et al., 2023). Further, abnormal triglyceride accumulation can contribute to oxidative stress and inflammatory responses within the kidneys (Au-Yeung et al., 2023). Elevated levels of triglycerides are also associated with an increased risk of cardiovascular diseases, such as atherosclerosis and coronary heart disease (Chait, 2022). In addition, VLDL and LDL may have adverse effects in kidney diseases. Elevated levels of VLDL and LDL are associated with glomerular lesions, interstitial fibrosis, and tubular cell damage, which can intensify the progression of kidney diseases and lead to albumin leakage into the urine (Wahl et al., 2016; Huang and Lee, 2022). Moreover, increased levels of VLDL and LDL may induce and aggravate renal inflammation, activating inflammatory cells and promoting immune cell migration (Wahl et al., 2016). These lipoproteins are also associated with an increased risk of atherosclerosis and coronary heart disease (Akers et al., 2019). HDL, with its reverse cholesterol transport function, facilitates the clearance of cholesterol from arterial walls and its transport to the liver for metabolism, thus lowering the risk of atherosclerosis and coronary heart disease (Ossoli et al., 2019). However, the role of HDL in kidney diseases is more complex. While HDL is generally considered to have a protective effect on the glomerular filtration barrier, in specific kidney diseases, elevated HDL levels may be associated with increased oxidative stress and inflammation in the glomerular basement membrane, leading to albumin leakage (Zhong et al., 2019). Several studies have suggested that higher HDL levels may be correlated with the activation of inflammatory cells and increased inflammation in kidney diseases (Strazzella et al., 2021). |
|  | c) | Clinical relevance: Discuss whether the results have clinical or public policy relevance, and to what extent they inform effect sizes of possible interventions | 7, 8 | In this study, we found differences in the risk of albuminuria associated with lipid metabolism disorders among different racial subgroups, particularly in Non-Hispanic Black individuals who exhibited an increased risk of developing the condition. Further, factors such as gender, age, education level, BMI, and the presence of comorbidities (e.g., hypertension, coronary heart disease, stroke, diabetes) also influence the individual's susceptibility to albuminuria risk in the context of lipid metabolism disorders. In clinical management, maintaining a healthy lipid metabolism may help prevent or alleviate the occurrence of microalbuminuria and related kidney diseases (Reidy and Ross, 2021). Given the differences in lipid metabolism among individual patients, personalized therapy plays a crucial role in clinical management. We suggest that, for patients at high risk of abnormal lipid metabolism and microalbuminuria, doctors should tailor specific treatment plans based on their unique conditions, incorporating lipid regulation, renal protection, and other measures to achieve more effective therapeutic outcomes. |
| 17 | **Generalizability** | Discuss the generalizability of the study results (a) to other populations, (b) across other exposure periods/timings, and (c) across other levels of exposure | 8 | Additionally, the MR analysis was limited to European populations and cannot be generalized to other ethnic groups. |
|  | **OTHER INFORMATION** |  |  |  |
| 18 | **Funding** | Describe sources of funding and the role of funders in the present study and, if applicable, sources of funding for the databases and original study or studies on which the present study is based | 9 | This study was funded by Natural Science Foundation of Zhejiang Province (LQ20H020002), General Research Project of Zhejiang Provincial Department of Education(Y201942047) and Wenzhou Science and Technology Program(Y20180060). |
| 19 | **Data and data sharing** | Provide the data used to perform all analyses or report where and how the data can be accessed, and reference these sources in the article. Provide the statistical code needed to reproduce the results in the article, or report whether the code is publicly accessible and if so, where | 8 | All data generated or analyzed in this study are available from published datasets. |
| 20 | **Conflicts of Interest** | All authors should declare all potential conflicts of interest | 8 | The authors have no competing interests to declare that are relevant to the content of this article. |

This checklist is copyrighted by the Equator Network under the Creative Commons Attribution 3.0 Unported (CC BY 3.0) license.

1. Skrivankova VW, Richmond RC, Woolf BAR, Yarmolinsky J, Davies NM, Swanson SA, et al. Strengthening the Reporting of Observational Studies in Epidemiology using Mendelian Randomization (STROBE-MR) Statement. JAMA. 2021;under review.

2. Skrivankova VW, Richmond RC, Woolf BAR, Davies NM, Swanson SA, VanderWeele TJ, et al. Strengthening the Reporting of Observational Studies in Epidemiology using Mendelian Randomisation (STROBE-MR): Explanation and Elaboration. BMJ. 2021;375:n2233.
