# Supplementary material for: Lipid metabolism disorders and albuminuria risk: insights from National Health and Nutrition Examination Survey 2001–2018 and Mendelian randomization analyses
Source: Ren Fail. 2024 Nov 3;46(2):2420841. doi: 10.1080/0886022X.2024.2420841 (PMC11536668; doi:10.1080/0886022X.2024.2420841)
Supplement: Supplementary Figure Legends.docx [file IRNF_A_2420841_SM2539.docx]

**Supplementary Figure Legends:**

**Figure S1.** Subgroup analysis for the association between triglycerides and albuminuria. Association between triglycerides and albuminuria by full-adjusted weighted multivariate logistic regression models. **A**: Forest plots showing association between triglycerides and albuminuria. **B**: Association between triglycerides and albuminuria. The odds ratio (OR) was estimated using the full-adjusted weighted multivariate logistic regression models. The horizontal bars represent 95% confidence intervals (CI). **C**: The P for interaction represents the results of the interaction tests.

**Figure S2.** Subgroup analysis for the association between triglycerides and albuminuria. Association between triglycerides and albuminuria by full-adjusted weighted multivariate logistic regression models. **A**: Forest plots showing association between triglycerides and albuminuria. **B**: Association between triglycerides and albuminuria. The odds ratio (OR) was estimated using the full-adjusted weighted multivariate logistic regression models. The horizontal bars represent 95% confidence intervals (CI). **C**: The P for interaction represents the results of the interaction tests.

**Figure S3.** Subgroup analysis for the association between HDL and albuminuria. Association between HDL and albuminuria by full-adjusted weighted multivariate logistic regression models. **A**: Forest plots showing association between HDL and albuminuria. **B**: Association between HDL and albuminuria. The odds ratio (OR) was estimated using the full-adjusted weighted multivariate logistic regression models. The horizontal bars represent 95% confidence intervals (CI). **C**: The P for interaction represents the results of the interaction tests.

**Figure S4.** Subgroup analysis for the association between cholesterol and albuminuria. Association between cholesterol and albuminuria by full-adjusted weighted multivariate logistic regression models. **A**: Forest plots showing association between cholesterol and albuminuria. **B**: Association between cholesterol and albuminuria. The odds ratio (OR) was estimated using the full-adjusted weighted multivariate logistic regression models. The horizontal bars represent 95% confidence intervals (CI). **C**: The P for interaction represents the results of the interaction tests.

**Figure S5.** Scatter plots for MR analyses of the causal effect of lipid metabolism on albuminuria. The MR analyses were carried out utilizing various methods, including fixed-effect inverse variance weighting, weighted mean, MR-Egger, Weight mode, and Weight median. Each line's slope represents the estimated MR effect for the specific method, with error bars indicating the 95% confidence intervals around each SNP. **A:** Cholesterol esters in large HDL. **B:** Cholesterol esters in large VLDL. **C:** Cholesterol esters in medium HDL. **D:** Cholesterol esters in medium LDL. **E:** Cholesterol esters in medium VLDL. **F:** Chylomicrons and extremely large VLDL particles. **G:** HDL cholesterol levels. **H:** LDL cholesterol levels. **I:** Total cholesterol.

**Figure S6.** Scatter plots for MR analyses of the causal effect of triglycerides on albuminuria. The MR analyses were carried out utilizing various methods, including fixed-effect inverse variance weighting, weighted mean, MR-Egger, Weight mode, and Weight median. Each line's slope represents the estimated MR effect for the specific method, with error bars indicating the 95% confidence intervals around each SNP.

**Figure S7.** Funnel plots for the causal effect of lipid metabolism on albuminuria. Funnel plots were generated for Mendelian randomization (MR) analyses investigating the relationship between lipid metabolism and albuminuria. These plots display the inverse variance weighted MR estimate for each single-nucleotide polymorphism with cytokines plotted against 1/standard error (1/SEIV). **A:** Cholesterol esters in large HDL. **B:** Cholesterol esters in large VLDL. **C:** Cholesterol esters in medium HDL. **D:** Cholesterol esters in medium LDL. **E:** Cholesterol esters in medium VLDL. **F:** Chylomicrons and extremely large VLDL particles. **G:** HDL cholesterol levels. **H:** LDL cholesterol levels. **I:** Total cholesterol.

**Figure S8.** Funnel plots for the causal effect of triglycerides on albuminuria.

Funnel plots were generated for Mendelian randomization (MR) analyses investigating the relationship between triglycerides and albuminuria. These plots display the inverse variance weighted MR estimate for each single-nucleotide polymorphism with cytokines plotted against 1/standard error (1/SEIV).

**Figure S9.** Plots of leave-one-out analyses for the causal effect of lipid metabolism on albuminuria. Forest plots illustrating the causal estimates of lipid metabolism on albuminuria by sequentially excluding each instrumental variable. The horizontal bars depict the beta value and its corresponding 95% confidence intervals (CI) for each causal estimate. **A:** Cholesterol esters in large HDL. **B:** Cholesterol esters in large VLDL. **C:** Cholesterol esters in medium HDL. **D:** Cholesterol esters in medium LDL. **E:** Cholesterol esters in medium VLDL. **F:** Chylomicrons and extremely large VLDL particles. **G:** HDL cholesterol levels. **H:** LDL cholesterol levels. **I:** Total cholesterol.

**Figure S10.** Plots of leave-one-out analyses for the causal effect of triglycerides on albuminuria. Forest plots illustrating the causal estimates of triglycerides on albuminuria by sequentially excluding each instrumental variable. The horizontal bars depict the beta value and its corresponding 95% confidence intervals (CI) for each causal estimate.
